# Metabolic engineering of *Pseudomonas bharatica* CSV86^T^ to degrade Carbaryl (1-naphthyl-*N*-methylcarbamate) via the salicylate-catechol route

**DOI:** 10.1128/spectrum.00284-24

**Published:** 2024-06-13

**Authors:** Harshit Malhotra, Tushar Dhamale, Sukhjeet Kaur, Sravanti T. Kasarlawar, Prashant S. Phale

**Affiliations:** 1 Department of Biosciences and Bioengineering, Indian Institute of Technology-Bombay, Mumbai, India; Migal-Galilee Research Institute, Kiryat Shmona, Israel

**Keywords:** bioremediation, Carbaryl, metabolic engineering, carbon source utilization hierarchy, transporter, metabolic analysis

## Abstract

*Pseudomonas bharatica* CSV86^T^ displays the unique property of preferential utilization of aromatic compounds over simple carbon sources like glucose and glycerol and their co-metabolism with organic acids. Well-characterized growth conditions, aromatic compound metabolic pathways and their regulation, genome sequence, and advantageous eco-physiological traits (indole acetic acid production, alginate production, fusaric acid resistance, organic sulfur utilization, and siderophore production) make it an ideal host for metabolic engineering. Strain CSV86^T^ was engineered for Carbaryl (1-naphthyl-*N*-methylcarbamate) degradation via salicylate-catechol route by expression of a Carbaryl hydrolase (CH) and a 1-naphthol 2-hydroxylase (1NH). Additionally, the engineered strain exhibited faster growth on Carbaryl upon expression of the McbT protein (encoded by the *mcb*T gene, a part of Carbaryl degradation upper operon of *Pseudomonas* sp. C5pp). Bioinformatic analyses predict McbT to be an outer membrane protein, and Carbaryl-dependent expression suggests its probable role in Carbaryl uptake. Enzyme activity and protein analyses suggested periplasmic localization of CH (carrying transmembrane domain plus signal peptide sequence at the N-terminus) and 1NH, enabling compartmentalization of the pathway. Enzyme activity, whole-cell oxygen uptake, spent media analyses, and qPCR results suggest that the engineered strain preferentially utilizes Carbaryl over glucose. The plasmid-encoded degradation property was stable for 75–90 generations even in the absence of selection pressure (kanamycin or Carbaryl). These results indicate the utility of *P. bharatica* CSV86^T^ as a potential host for engineering various aromatic compound degradation pathways.

**IMPORTANCE:**

The current study describes engineering of Carbaryl metabolic pathway in *Pseudomonas bharatica* CSV86^T^. Carbaryl, a naphthalene-derived carbamate pesticide, is known to act as an endocrine disruptor, mutagen, cytotoxin, and carcinogen. Removal of xenobiotics from the environment using bioremediation faces challenges, such as slow degradation rates, instability of the degradation phenotype, and presence of simple carbon sources in the environment. The engineered CSV86-MEC2 overcomes these disadvantages as Carbaryl was degraded preferentially over glucose. Furthermore, the plasmid-borne degradation phenotype is stable, and presence of glucose and organic acids does not repress Carbaryl metabolism in the strain. The study suggests the role of outer membrane protein McbT in Carbaryl transport. This work highlights the suitability of *P. bharatica* CSV86^T^ as an ideal host for engineering aromatic pollutant degradation pathways.

## INTRODUCTION

Agriculture sector consumes pesticides (insecticides, fungicides, herbicides, etc.) on a large scale for crop protection and to increase the production to fulfil ever-growing demand for food. Besides killing the target pests, these xenobiotics get distributed into the environment impacting non-target biota including humans (1, 2). Carbaryl (1-naphthyl-*N*-methylcarbamate, CAS No. 63-25-2, a naphthalene-based carbamate pesticide), a widely used carbamate family pesticide, is highly toxic (depending on the concentration and organism) to target as well as non-target biota (like earthworms and honeybees) and has been classified as a potential human carcinogen (3, 4). Due to its widespread distribution in the environment, various microbes have evolved and adapted different metabolic routes for its degradation. Examples include *Arthrobacter* sp. RC100 (5), *Micrococcus* sp. (6), *Pseudomonas* sp. strains C4, C5pp, C6, C7 (7, 8), *Burkholderia* sp. C3 (9), *P. putida* XWY-1 (10), among others (4).

Bioremediation using microbes has been proposed to be a desirable alternative to abiotic removal of xenobiotics (11–15). However, application of natural isolates poses limitations, such as inefficient degradation, poor survival due to abiotic stress, limited metabolic diversity, repression of xenobiotic metabolism in the presence of simple carbon sources, among others (15–18). These limitations can be resolved by application of recombinant DNA techniques to construct “patchwork assembly” of various pathways into a single organism/host, referred to as metabolic engineering. An ideal host for such assembly should exhibit various traits, such as well-characterized genome sequence and growth conditions, stress tolerance as well as availability of host-specific genetic manipulation tools, among others (19). The heterologous expression of degradative enzymes can either be plasmid or chromosome based. For example, the pSEVA plasmids (shuttle vectors) are widely used expression vectors for metabolic engineering of *Pseudomonas* spp. (20). Whereas, chromosomal integration can be carried out using homologous recombination, transposons, or CRISPR/Cas9-based vectors for phenotypic stability (19). “Patchwork assembly” approaches have been employed to engineer strains for degradation of aromatic pollutants through diverse pathways. For example, *Pseudomonas* spp. have been engineered for chloro- and methyl-aromatic compound catabolism (21), *P. putida* KT2440 for carbofuran hydrolysis (22), *Azoarcus communis* SWub3 for anaerobic benzoate degradation (23), *Escherichia coli* for 4-fluorophenol mineralization (24), *P. putida* KTU for 1,2-dichloroethane degradation (25), among others. These approaches have also been combined with directed evolution of degradative enzymes and proteins to enhance the substrate range and degradation efficiency (26).

*Pseudomonas bharatica* CSV86^T^, an Indian soil bacterium isolated from petroleum product-contaminated soil, degrades a wide range of mono- and poly-cyclic aromatic hydrocarbons efficiently (12–14 h) at high concentrations (up to 1% wt/vol) through diverse metabolic routes (27, 28). Strain CSV86^T^ exhibits the novel property to metabolize aromatic compounds preferentially over simple carbon sources like glucose and glycerol, and co-metabolize them with organic acids (28–30). This trait makes CSV86^T^ unique among various reported *Pseudomonas* spp., which exhibit carbon catabolite repression of aromatic compound utilization in the presence of both organic acids and glucose (31). The carbon catabolite repression in *Pseudomonas* spp. is primarily orchestrated by CbrA/CbrB-Hfq/Crc-*crc*Z system, wherein the Crc-Hfq complex binds the mRNA of secondary carbon source utilization genes, thereby repressing translation in the presence of preferred carbon sources. The *crc*Z (or its homologs *crc*Y or *crc*X) small RNA sequesters the Crc-Hfq complex to relieve repression. The expression of these small RNAs is controlled by the CbrA/CbrB two-component regulatory system, although the underlying signals are unknown (32). However, the specific regulatory mechanisms responsible for the unique preferential utilization phenotype of strain CSV86^T^ remain elusive.

Additionally, well-studied aromatic compound metabolic pathways and their regulation, growth conditions, genome sequence, plasmid-free strain, stable degradation phenotype, and various eco-physiological traits like heavy metal resistance, indole acetic acid production, alginate production, fusaric acid resistance, organic sulfur utilization, and siderophore production make strain CSV86^T^ an ideal host for metabolic engineering (27–29, 33–36).

**Fig 1:**
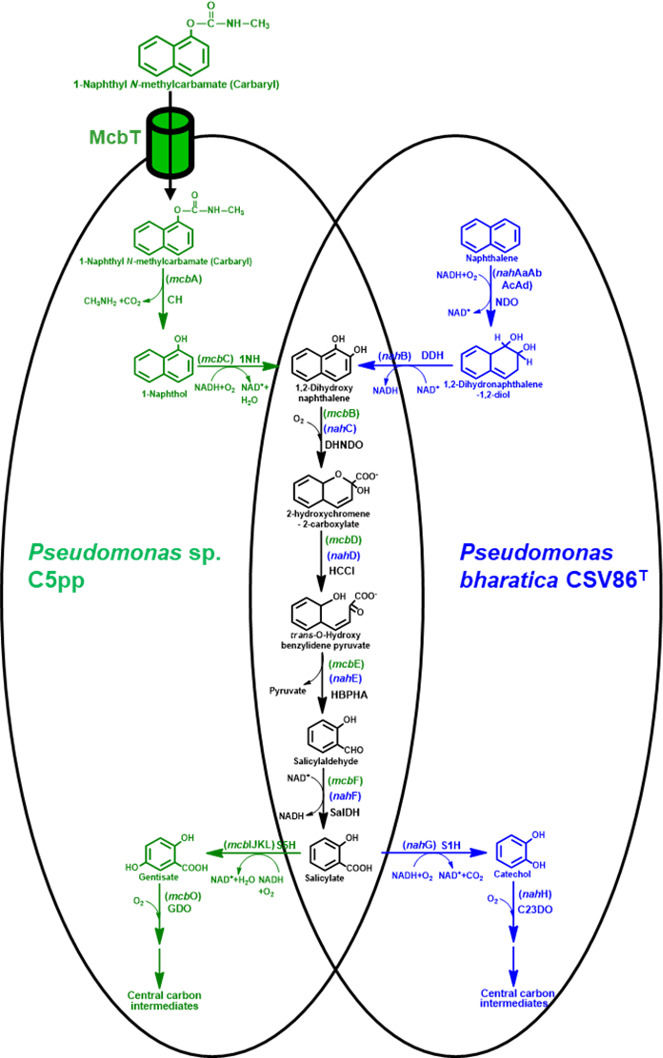
Metabolic pathways for aerobic Carbaryl (1-naphthyl *N*-methylcarbamate) and naphthalene degradation in *Pseudomonas* sp. C5pp and *Pseudomonas bharatica* CSV86^T^, respectively. The metabolic steps, intermediate metabolites, and enzymes depicted in the overlapping region represent common metabolic steps funneling the substrate into central carbon pathway. The pathway intermediates, genes (in brackets), and enzymes/proteins are indicated in green for strain C5pp and in blue for CSV86^T^. The enzyme/protein abbreviations are McbT, probable Carbaryl transporter; CH, Carbaryl hydrolase; 1NH, 1-naphthol 2-hydroxylase; DHNDO, 1,2-dihydroxynaphthalene dioxygenase; HCCI, 2-hydroxychromene-2-carboxylate isomerase; HBPHA, *trans-O*-hydroxybenzylidene pyruvate hydratase-aldolase; SalDH, salicylaldehyde dehydrogenase; S5H, salicylate-5-hydroxylase; GDO, gentisate-1,2-dioxygenase; NDO, naphthalene dioxygenase; DDH, *cis*-dihydrodiol dehydrogenase; S1H, salicylate-1-hydroxylase; C23DO, catechol-2,3-dioxygenase.

The metabolism of naphthalene in *P. bharatica* CSV86^T^ is initiated by the concerted action of naphthalene dioxygenase and *cis*-dihydrodiol dehydrogenase to form 1,2-dihydroxynaphthalene, which is subsequently metabolized to salicylate. Furthermore, salicylate-1-hydroxylase converts salicylate to catechol, which is ring cleaved by the action of catechol-2,3-dioxygenase (C23DO) to yield aliphatic intermediate that enters the central carbon pathway (33, 37; Fig. 1). Genes encoding naphthalene degradation enzymes are present as two operons (*nah* and *sal* operon; 13 kb apart) on an integrative and conjugative element ICE*nah*CSV86 in strain CSV86^T^ (34; Fig. S1A). The *nah* operon (*nah*AaAbAcAdBFCED) consists of genes coding for enzymes involved in the metabolism of naphthalene to salicylate. Whereas, the *sal* operon (*nah*GTHINLOMKJX) consists of genes encoding enzymes involved in the conversion of salicylate to central carbon intermediates. The transcription of *nah* operon is driven by the promoter P*nah* while that of *sal* operon is driven by P*sal* (34, 38; Fig. S1A).

The bacterial degradation of Carbaryl is initiated by the action of Carbaryl hydrolase (CH) to generate 1-naphthol, a highly toxic metabolic intermediate (39). 1-Naphthol is further hydroxylated by 1-naphthol-2-hydroxylase (1NH) to yield 1,2-dihydroxynaphthalene. This intermediate is metabolized to salicylate through a series of enzymatic steps and funneled into central carbon pathway either through catechol or gentisate route (4). Based on biochemical evidence, it is proposed that Carbaryl degradation in *Pseudomonas* sp. C5pp proceeds via formation of 1-naphthol, which is converted to 1,2-dihydroxynaphthalene and further, salicylate. The action of salicylate-5-hydroxylase generates gentisate, which is ring cleaved to aliphatic intermediates by the action of gentisate dioxygenase (GDO) (7; Fig. 1). Genes encoding enzymes for the conversion of Carbaryl via 1,2-dihydroxynaphthalene to salicylate are arranged as Carbaryl degradation “upper operon” (40, 41; Fig. S1B). The presence of common metabolic steps involved in Carbaryl and naphthalene degradation (Fig. 1) can be exploited to increase the metabolic diversity of strain CSV86^T^ by pathway engineering.

In the present study, strain CSV86^T^ was engineered to metabolize Carbaryl via salicylate-catechol route by expressing CH, 1NH, and the putative Carbaryl transporter, McbT from *Pseudomonas* sp. C5pp (Fig. 1). The engineered strain was characterized at biochemical and molecular level for Carbaryl metabolism, phenotypic stability, and preferential utilization property.

## RESULTS AND DISCUSSION

### Co-expression and localization of Carbaryl hydrolase and 1-naphthol 2-hydroxylase

The metabolic steps from 1,2-dihydroxynaphthalene to salicylate are common for naphthalene degradation in strain CSV86^T^ and Carbaryl degradation in strain C5pp (Fig. 1; Table 1). The promoter P*nah* from CSV86^T^ was found to be leaky and induced to high levels using naphthalene or salicylate, indicating it to be a strong promoter (using construct pSEVAP*nah*-1NH derived from pSEVA234; Table 2) (38). This promoter was used to engineer CSV86^T^ for Carbaryl degradation. In Carbaryl-degrading soil isolate *Pseudomonas* sp. C5pp, the presence of 96-amino-acid-long N-terminus transmembrane domain plus signal peptide (Tmd + Sp) region as a part of Carbaryl hydrolase enzyme (encoded by *mcb*A) aids in localizing it (~72 kDa, mature CH) to the periplasmic space (42). We constructed pMEC1 (P*nah*→CH-1NH), which harbors full-length *mcb*A (encoding CH carrying Tmd + Sp at N-terminus to localize mature CH to periplasm and 6× His tag at C-terminus) and *mcb*C (encoding 1NH) under the P*nah* promoter (see Materials and Methods for details; Table 2; Fig. S2A).

**TABLE 1:** List of microbes and engineered strains used in the study

Strain	Relevant genotype	Relevant growth properties^*a*^	Preferential utilization trait	Reference
*Escherichia coli* DH5α	*sup*E44, Δ*lac*U169(ϕ80 *lac*ZΔM15), *hsdR*17, *rec*A1, *end*A1, *gyr*A96, *rel*A1	NA	NA	Novagen
*Pseudomonas* sp. C5pp	*mcb*ATBCDEF (Carbaryl upper operon), *mcb*IJKL (Carbaryl middle operon), *mcb*OPQ (Carbaryl lower operon)	Carbaryl^+^, 1-naphthol^+^, salicylate^+^, gentisate^+^, naphthalene^−^	Glucose preferred over aromatics	(7, 41)
*P. bharatica* CSV86^T^	*nah*AaAbAcAdBFCED (naphthalene upper operon), *nah*GTHINLOMKJX (naphthalene lower operon)	Naphthalene^+^, salicylate^+^, Carbaryl^−^, 1-naphthol^−^, gentisate^−^	Aromatics preferred over glucose	(28, 33)
CSV86-MEC1 (*P. bharatica* CSV86^T^ harboring pMEC1)	*nah*AaAbAcAdBFCED (naphthalene upper operon), *nah*GTHINLOMKJX (naphthalene lower operon), *mcb*AC (plasmid encoded)	Carbaryl^+^, 1-naphthol^+^, naphthalene^+^, salicylate^+^, gentisate^−^	Aromatics preferred over glucose	Present study
CSV86-MEC2 (*P. bharatica* CSV86^T^ harboring pMEC2)	*nah*AaAbAcAdBFCED (naphthalene upper operon), *nah*GTHINLOMKJX (naphthalene lower operon), *mcb*ATC (plasmid encoded)	Carbaryl^+^, 1-naphthol^+^, naphthalene^+^, salicylate^+^, gentisate^−^	Aromatics preferred over glucose	Present study

^*a*^ + indicate growth on the substrate, − indicates no growth, and NA indicates not applicable.

**TABLE 2:** List of plasmids and primers used in the study

Name	Characteristics or sequence (5′−3′)^*a*^		Reference
Plasmids			
pSEVA234	Shuttle expression vector; *oriV*(pBBR1), *lacI*^Q^ P*trc*, *aph*A, Km^R^		43
pSEVAP*nah*-1NH	Expression vector (derived from pSEVA234) carrying 1NH under the P*nah* promoter; *oriV*(pBBR1) P*nah*→1NH *aph*A, Km^R^		38
pMEC1 (pSEVAP*nah*CH-1NH)	Expression vector (derived from pSEVA234) carrying CH and 1NH under the P*nah* promoter; *oriV*(pBBR1), P*nah*→CH-1NH, *aph*A, Km^R^		Present study
pMEC2 (pSEVAP*nah*CH-McbT-1NH)	Expression vector (derived from pSEVA234) carrying CH, McbT and 1NH under the P*nah* promoter; *oriV*(pBBR1), P*nah*→CH-McbT-1NH, *aph*A, Km^R^		Present study
Primers			
Cloning and expression			
CH-FP	TTGGGGTACC*AGAGGAGGAAAA*ATGGCGGTCACGGCAAATTATTTGC (*Kpn*I)		
CH-RP	TAATGGATCCTCA**ATGATGATGATGATGATG**CGCGGCAAGCCGGTCG (*Bam*HI)		
*mcb*T-FP	TAATGGATCC*AGAGGAGGAAAA*ATGAAAACCTGCTCATTCGCCG (*Bam*HI)		
*mcb*T-RP	CGCCTCTAGACTAGAGCGGGAAAACGGCCC (*Xba*I)		
Co-transcription primers for Carbaryl upper operon			
*mcb*A-*mcb*T	F_AT1	GCTCCGGCAACGAATTGATCG	
	R_AT1	GCGTGGATGATCAGACGTGTGAC	
*mcb*T-*mcb*B	F_TB1	GAGCTGGTTAAGCGAGGTGATGTCG	
	R_TB1	CGTTGACACCTAGCGCGTAGAGG	
*mcb*B-*mcb*C	F_BC1	GCTTGGTTGAGGAGTGCGGC	
	R_BC1	CCCTTTTGATCCCAAGCCATTCTTCC	
*mcb*C-*mcb*D	F_CD1	GGGGGGTAGGGCAGGCG	
	R_CD1	CGAGTGCACCAGACAACATTTTCCATC	
*mcb*D-*mcb*E	F_DE1	GACACCGTTAAAACTGCGTGATGCTG	
	R_DE1	CTACCAACATTGAGGCTTCTCGAATTG	
*mcb*E-*mcb*F	F_EF1	CTGTATTTGGTCGCGATATTAGCAATGC	
	R_EF1	CTATAAAATCACGCTTTTCATCCCAGGTC	
*mcb*A-*mcb*T-*mcb*B	F_TB1	GAGCTGGTTAAGCGAGGTGATGTCG	
	R_AT1	GCGTGGATGATCAGACGTGTGAC	
*mcb*T-*mcb*B-*mcb*C	F_BC1	GCTTGGTTGAGGAGTGCGGC	
	R_TB1	CGTTGACACCTAGCGCGTAGAGG	
*mcb*C-*mcb*D-*mcb*E	F_DE1	GACACCGTTAAAACTGCGTGATGCTG	
	R_CD1	CGAGTGCACCAGACAACATTTTCCATC	
*mcb*C-*mcb*D-*mcb*E-*mcb*F	F_EF1	CTGTATTTGGTCGCGATATTAGCAATGC	
	R_CD1	CGAGTGCACCAGACAACATTTTCCATC	
Quantitative PCR			
*nah*Aa-FP	CGCGATGCTACAGTTCAGTCC		
*nah*Aa-RP	GCAGATAGGCCGTACCAAGAGG		
*nah*G-FP	CGAGCACTTGGTGGATGTCC		
*nah*G-RP	CGGGATGCACTCCAGTAAGG		
*zwf*-FP	GGACACCACGGTGTTGC		
*zwf*-RP	CGCAGTTCTTCCCGGAA		
			

^*a*^ Km^R^ represents kanamycin resistance. In the nucleotide sequence, restriction sites (in parentheses) are indicated by underlining, ribosome-binding sites are indicated in italics, and 6× His tTag is marked in bold.

To assess the functionality of pMEC1 in strain CSV86^T^ (transformant referred as CSV86-MEC1), expression and localization of CH and 1NH were monitored by measuring enzyme activity, SDS-PAGE, and Western blot analysis. The activities of CH, 1NH, and ring-cleaving enzyme (catechol-2,3-dioxygenase) were monitored from the periplasmic, cytoplasmic, and cell-free extract (CFE; which contains both cytoplasmic and periplasmic proteins) fractions prepared from CSV86-MEC1 cells grown on naphthalene. Naphthalene was chosen as the growth substrate for CSV86-MEC1 for this analysis as it is the native inducer of the P*nah* promoter (38). Subsequently, naphthalene-grown cells were used as inoculum for carrying out growth, metabolic, and biochemical analyses of the engineered strain. The strain CSV86^T^ naturally degrades naphthalene, and various global-level adaptive responses occur while growing on naphthalene which might be advantageous for Carbaryl degradation by the engineered strain. Enzyme activities from naphthalene-grown wild-type CSV86^T^ (which lacks CH and 1NH enzymes) and Carbaryl-grown *Pseudomonas* sp. C5pp were taken as negative and positive control, respectively.

The CH activity was determined from various fractions to assess the functionality of the intrinsic N-terminus signal sequence, Tmd + Sp, to translocate CH to periplasmic space in strain CSV86-MEC1. The activity of 1NH (which lacks a signal sequence) was examined in various fractions to determine its localization, as certain aromatic hydroxylases of *Pseudomonas* spp., which lack a signal sequence, have been reported to be localized to the periplasm (44, 45). The activity of ring-cleaving dioxygenase C23DO [encoded by *nah*H gene of the naphthalene degradation lower operon in CSV86^T^ (*sal* operon)], which is reported to be cytoplasmic enzyme (38), was taken as a control to ensure proper fractionation of CSV86-MEC1 cells, i.e., no contamination of cytoplasmic proteins in the periplasmic fraction. Similarly, the activity of the cytoplasmic enzyme gentisate dioxygenase (encoded by *mcb*O gene of Carbaryl degradation lower operon in C5pp) was taken as control to ensure efficient fractionation of C5pp cells (42).

The activity of CH in the periplasmic fraction was 1,058 nmol·min^−1^·mg^−1^ (~22-fold higher) compared to cytoplasmic fraction (48 nmol·min^−1^·mg^−1^), while the activity in CFE was 150 nmol·min^−1^·mg^−1^ (Table 3) in CSV86-MEC1. The conversion of Carbaryl to 1-naphthol by periplasmic fraction was also monitored by recording time-dependent spectral changes at 322 nm, which is λ_max_ for 1-naphthol (Fig. S3). Carbaryl-grown C5pp cells displayed ~7-fold higher CH activity (1,086 nmol·min^−1^·mg^−1^) in the periplasm as compared to cytoplasmic fraction (167 nmol·min^−1^·mg^−1^) and CFE (145 nmol·min^−1^·mg^−1^; Table 3). Whereas, wild-type CSV86^T^ cells (negative control) showed negligible activity of CH in all fractions, indicating lack of hydrolase (esterase) enzymes acting on Carbaryl.

**TABLE 3:** Specific activities of Carbaryl hydrolase, 1-naphthol 2-hydroxylase, gentisate-1,2-dioxygenase, and catechol-2,3-dioxygenase from various fractions prepared from CSV86-MEC1, *Pseudomonas* sp. C5pp, and *P. bharatica* CSV86^T^ cells

Organism (grown on)	Fraction	Specific activity (nmol min^−1^ mg^−1^)		
		CH	1NH	GDO/C23DO^*a*^
CSV86-MEC1 (naphthalene)	CFE	150 ± 18	731 ± 6	454 ± 38
	Cytoplasm	48 ± 11	715 ± 146	518 ± 93
	Periplasm	1,058 ± 121	1,659 ± 73	Tr^*c*^
*Pseudomonas* sp. C5pp (Carbaryl)	CFE	145 ± 22	500 ± 71	389 ± 156
	Cytoplasm	167 ± 65	516 ± 40	388 ± 81
	Periplasm	1,086 ± 210	1,667 ± 162	ND^*b*^
*P. bharatica* CSV86^T^ (naphthalene)	CFE	Tr^*c*^	Tr^*c*^	1,247 ± 19
	Cytoplasm	Tr^*c*^	Tr^*c*^	1,050 ± 276
	Periplasm	Tr^*c*^	Tr^*c*^	Tr^*c*^

^*a*^ GDO activity was monitored from strain C5pp while C23DO from strain CSV86^T^ and CSV86-MEC1.

^*b*^ ND, not detected.

^*c*^ Tr, trace; enzyme activity less than 30 nmol·min^−1^·mg^−1^ is reported as trace.

**Fig 2:**
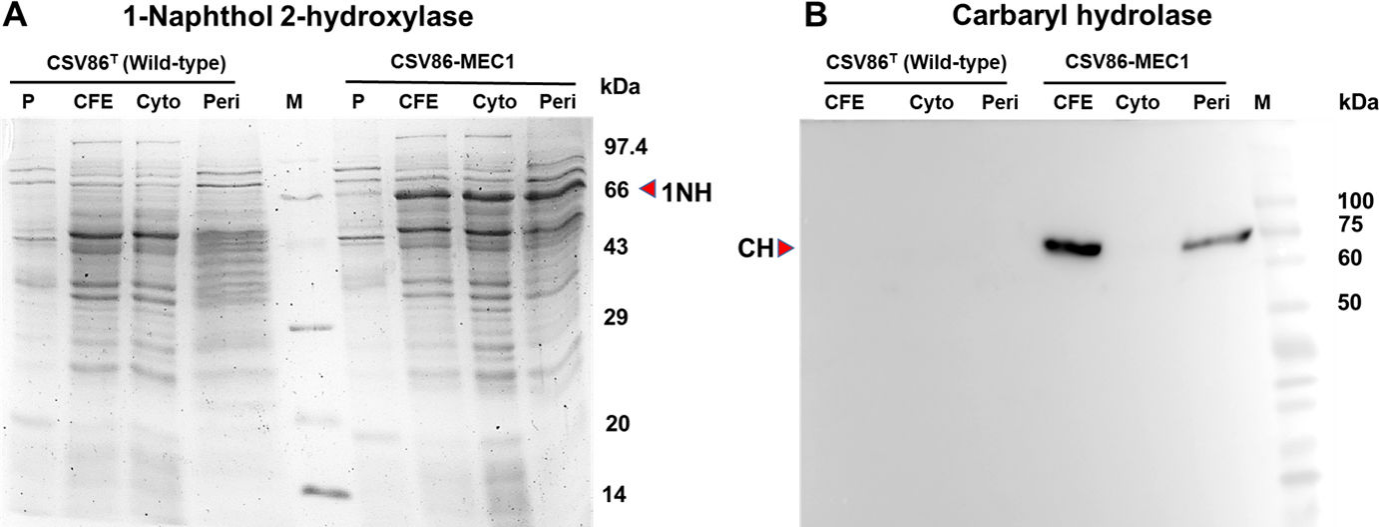
Expression and localization of 1-naphthol 2-hydroxylase and Carbaryl hydrolase in CSV86-MEC1: (**A**) SDS-PAGE analysis (12%) for the localization of 1NH (~66 kDa) and (**B**) Western blot analysis for the localization of CH (harboring 6× His tag at C-terminus; ~72 kDa) in CSV86-MEC1. “P” represents the pellet, “CFE” represents the cell-free extract, “Cyto” represents cytoplasmic, and “Peri” represents periplasmic fraction. “M” represents protein molecular weight markers. CH and 1NH bands are indicated by red arrow.

The SDS-PAGE analysis of these fractions from CSV86-MEC1 failed to show any detectable protein band corresponding to CH (~72 kDa; Fig. 2A). Western blot analysis using anti-His tag antibodies revealed the presence of immunoreactive protein band (~72 kDa, mature CH) in the periplasmic fraction as well as in the CFE (Fig. 2B). This indicates the cleavage of 96-amino-acid-long N-terminus Tmd + Sp region during translocation of CH to the periplasm in strain CSV86-MEC1 (Fig. 2B). These results corroborate the role of Tmd + Sp in translocation of CH to the periplasm as proposed earlier in strain C5pp (42). The immunoreactive band was not detectable in the cytoplasmic fraction, probably due to low amount of protein, as reflected by low CH activity (see enzyme activity in Table 3).

Regarding 1NH activity from CSV86-MEC1, intriguingly the periplasmic fraction (1,659 nmol·min^−1^·mg^−1^) displayed an ~2.3-fold increase as compared to cytoplasmic fraction (715 nmol·min^−1^·mg^−1^) and CFE (731 nmol·min^−1^·mg^−1^; Table 3). Carbaryl-grown C5pp cells showed ~3.2-fold higher activity of 1NH in the periplasm (1,667 nmol·min^−1^·mg^−1^) as compared to cytoplasmic fraction (516 nmol·min^−1^·mg^−1^) and CFE (500 nmol·min^−1^·mg^−1^; Table 3). Wild-type strain CSV86^T^ (negative control) displayed negligible activity of 1NH in all fractions, indicating lack of enzymes responsible for hydroxylation of 1-naphthol. SDS-PAGE analysis showed a distinct protein band (~66 kDa), corresponding to the molecular weight of 1NH in all fractions of CSV86-MEC1. The absence of this protein band in wild-type CSV86^T^ suggests it to be 1NH (Fig. 2A).

The activity of C23DO from CSV86-MEC1 and wild-type CSV86^T^ as well as GDO from strain C5pp was found to be present only in the cytoplasmic fraction and CFE, as compared to the periplasmic fraction (Table 3). These results indicate that the periplasmic fractionation of the cells by cold-osmotic shock treatment was efficient, suggesting no leakage/contamination of cytoplasmic enzymes due to rupture of inner membrane.

These observations indicate that 1NH, previously reported to be cytoplasmic in strain C5pp (42), was also found to be localized in the periplasm, despite absence of a signal peptide. Aromatic hydroxylases (*para*-cresol methyl hydroxylase from *P. putida* NCIMB 9869 and 4-ethylphenol methylene hydroxylase from *P. putida* JD19), which lack a conventional signal peptide, have been reported to be localized in the periplasmic space (44, 45). The periplasmic localization of 1NH is intriguing as the enzyme requires NAD(P)H cofactor to function, and the periplasm is an oxidizing environment. However, certain NADH-dependent enzymes such as NADH peroxidase (46), sulfite reductase (47), and glyceraldehyde-3-phosphate dehydrogenase (48) are also found to occur in the periplasmic space. The presence of periplasmic 1NH might play a role in scavenging 1-naphthol from the periplasmic space, mitigating its toxicity.

These results suggest the successful expression of CH and 1NH under P*nah* in CSV86-MEC1, indicating the functionality of pMEC1. CH and 1NH were found to be present in the periplasm of CSV86-MEC1 and C5pp, and expression levels were similar in both strains.

### Identification of an ORF encoding a putative outer membrane protein, McbT

**Fig 3:**
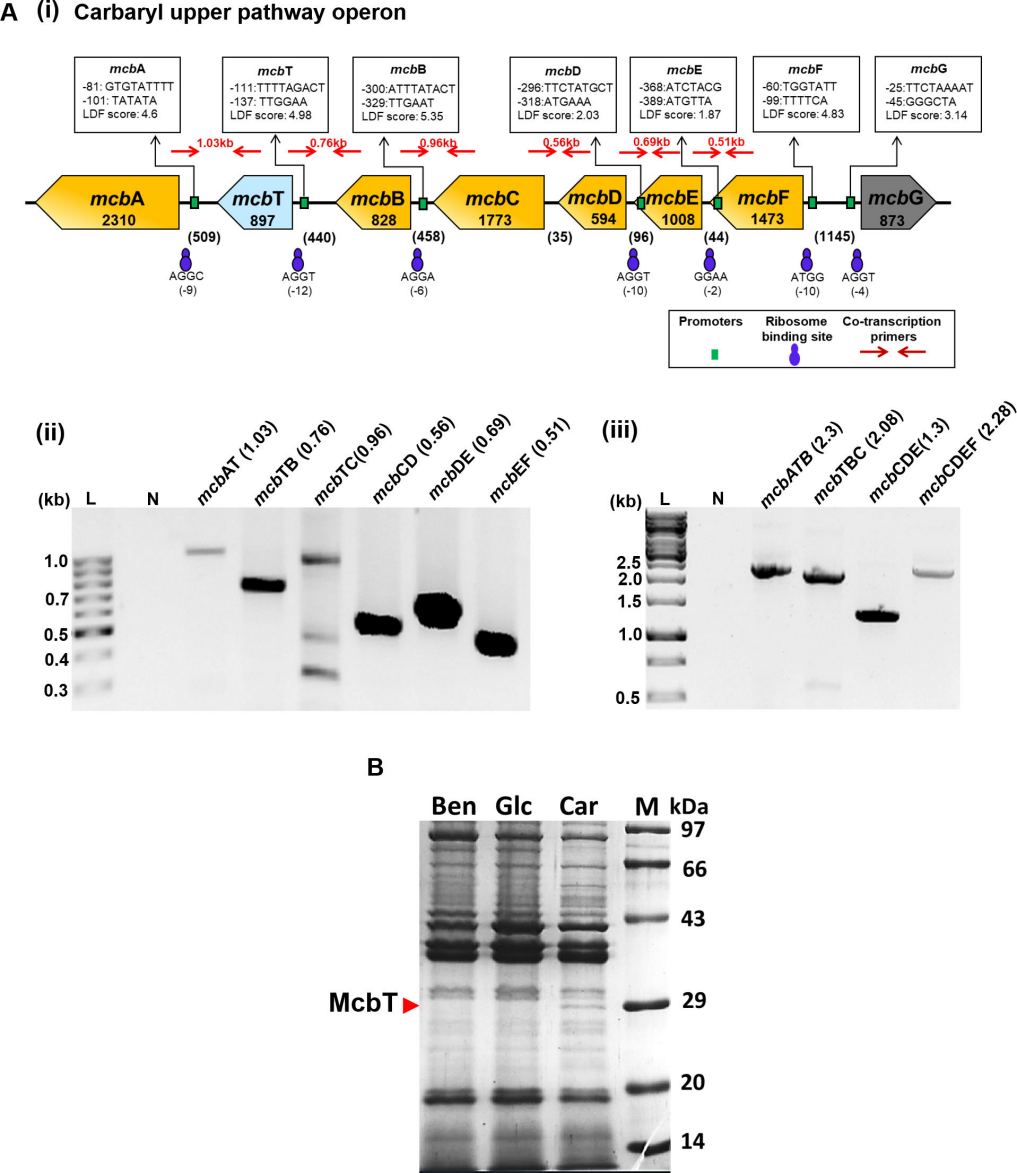
*In silico* analysis, co-transcription studies, and carbon-source-dependent expression of McbT in *Pseudomonas* sp. C5pp. (**A**) Gene arrangement, promoters, and co-transcription analysis of the Carbaryl upper pathway operon in *Pseudomonas* sp. strain C5pp. (i) Genes (name and length [bp]) are depicted in the orange box arrows. The newly annotated gene *mcb*T is indicated by blue box arrow. The regulatory gene *mcb*G is indicated by gray box arrow. The numbers in the parentheses indicate the intergenic distance in base pairs. The small green boxes depict putative promoters (with −10 box, −35 box, and LDF scores), whereas blue circles indicate ribosome-binding sites. Co-transcription primers are depicted in red arrows. Note: figure is not to the scale. Co-transcription analysis: electrophoretogram of amplicons (sizes indicated in kb) of (ii) two- or (iii) three- and four-gene pairs of the Carbaryl upper pathway operon. “L” indicates DNA ladder (100 bp); “N” indicates negative control. (**B**) SDS-PAGE (12%) analysis of outer membrane protein fraction prepared from *Pseudomonas* sp. C5pp cells grown on benzoate (Ben), glucose (Glc), and Carbaryl (Car). “M” denotes protein molecular weight markers (kDa). The protein band corresponding to McbT (~30 kDa) is marked by red arrow.

An open reading frame of 897 bp (rc13993-14889 in Supercontig-A in *Pseudomonas* sp. strain C5pp [41]), referred to as *mcb*T, was identified upon reannotation and found to be located in between *mcb*A (encodes CH) and *mcb*B (encodes 1,2-dihydroxynaphthalene dioxygenase) (Fig. 3Ai). *In silico* promoter prediction of upper operon indicated presence of multiple promoter elements (as indicated by linear discriminant function or LDF scores) (Fig. 3Ai). To validate *in silico* prediction, co-transcription analysis was performed using cDNA prepared from Carbaryl-grown C5pp cells as template and intergenic primers for two-, three-, or four-gene pairs (Table 2). The results are depicted in Fig. 3Aii and iii. The presence of amplicons of expected length indicates that the upper pathway genes (including *mcb*T) are co-transcribed as a polycistron.

The *mcb*T (897 bp) was translated *in silico* to 298-amino-acid-long polypeptide (referred as McbT) and showed sequence homology with characterized as well as uncharacterized outer membrane proteins involved in transport. BLAST analysis revealed that McbT showed homology with uncharacterized transporters from *Pseudomonas, Cupriavidus,* and *Burkholderia* spp. McbT exhibited 31.4%, 26.9%, and 21.4% identity with the functionally characterized hydrophobic compound transporters SphA [for sphingosine transport in *P. aeruginosa* PAO1 (49)], TcpY [for trichlorophenol transport in *C. necator* JMP134 (50)], and Pput2725 [for small hydrophobic molecule transport in *P. putida* F1, PDB ID: 4RL8 (51)], respectively. N-terminus of McbT harbors a signal peptide (located from 1 to 24 amino acid) with prediction probability of 0.97 and a cleavage site between A24 and T25 position (Fig. S4A). The mature McbT was expected to be 274 amino acid long with a theoretical molecular weight of 29.3 kDa. The secondary structure analysis predicted it to possess 12 contiguous β-strands, a characteristic feature of bacterial outer membrane proteins (52). The three-dimensional structure model constructed for McbT using AlphaFold2.0 has 14 stranded β-barrel structure with N-terminal loop occluding the barrel lumen from the periplasmic side (Fig. S4B and S4C), a characteristic feature of bacterial outer membrane proteins involved in the uptake of hydrophobic compounds (51). The SDS-PAGE analysis of outer membrane fractions prepared from Carbaryl-grown cells of strain C5pp showed a distinct protein band (~30 kDa), corresponding to the molecular weight of mature McbT (upon cleavage of the N-terminus signal peptide). The absence of this protein band in cells grown on benzoate or glucose strongly indicates that it is likely to be McbT, induced by Carbaryl (Fig. 3B).

The lipid asymmetry of the outer membrane and hydrophilic channels of porins in gram-negative bacteria prevent the entry of hydrophobic compounds like Carbaryl. However, some uncharged aromatic compounds can still passively diffuse through the membrane (53). Specific outer membrane proteins facilitate the uptake of certain hydrophobic compounds, such as CymD for cymene, TodX for toluene, SphA for sphingosine, and TcpY for trichlorophenol. The genes encoding these outer membrane proteins were also found to be present within their respective degradation operons (49, 50, 54). In strain C5pp, *mcb*T is present in the upper Carbaryl pathway operon and is co-transcribed with neighboring genes as *mcb*FEDCBTA to encode outer membrane protein McbT only in the presence of Carbaryl. Thus, based on *in silico*, co-transcription, and expression analyses, we propose McbT to be involved in the transport of Carbaryl across the outer membrane in strain C5pp. In order to assess the impact of McbT on growth on Carbaryl, *mcb*T was cloned into pMEC1 (P*nah*→CH-1NH), to generate construct pMEC2 (P*nah*→CH-McbT-1NH; see Materials and Methods for details; Table 2; Fig. S2B).

### Growth kinetics and metabolic analysis of engineered *P. bharatica* CSV86^T^

**Fig 4:**
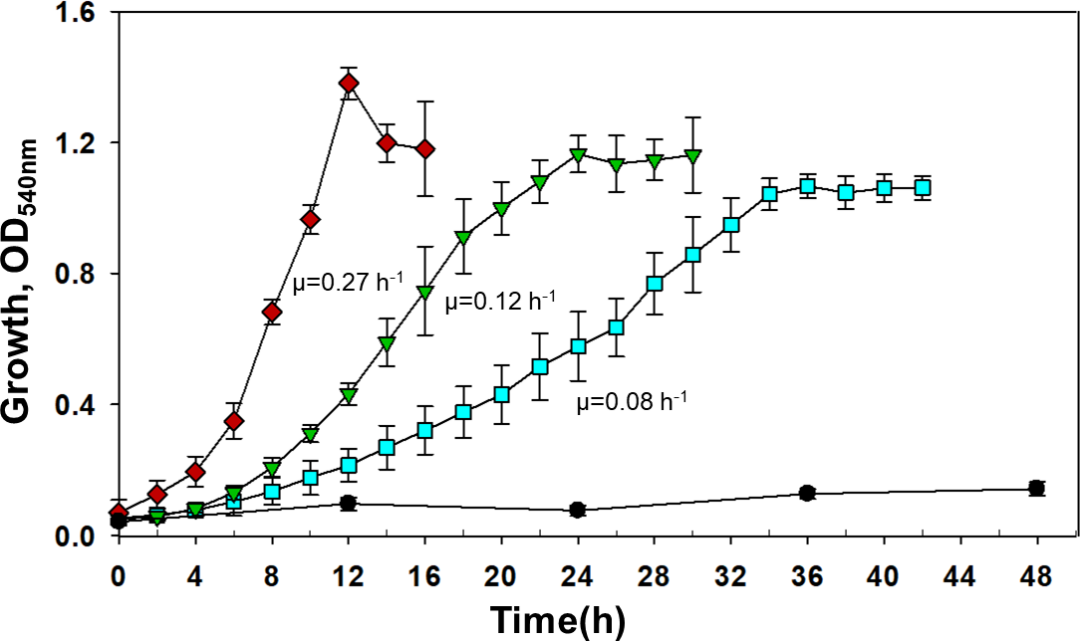
Growth profiles of *Pseudomonas* sp. strain C5pp (♦), *P. bharatica* CSV86^T^ (●), CSV86-MEC1 (■), and CSV86-MEC2 (▼) on Carbaryl (0.1%). The mean value with standard deviation of at least three independent experiments performed in duplicates is depicted.

The expression of CH and 1NH under P*nah* (pMEC1) in strain CSV86^T^ (CSV86-MEC1) rendered it capable of utilizing Carbaryl as the sole source of carbon and energy (Fig. 4). In order to assess the impact of McbT on growth, growth kinetics of CSV86-MEC1 and CSV86-MEC2 on Carbaryl were compared. *Pseudomonas* sp. C5pp and wild-type CSV86^T^ were used as controls. The specific growth rate (μ, h^−1^) of CSV86-MEC1 on Carbaryl was 0.08 h^−1^ with a long lag phase (6–8 h). However, the growth was slower than strain C5pp on Carbaryl (μ **=** 0.27 h^−1^). Whereas, CSV86-MEC2 (CSV86^T^ carrying pMEC2), which encodes McbT, CH, and 1NH under P*nah*, exhibited a growth profile with a shorter lag phase (4 h) and higher specific growth rate of 0.12 h^−1^ as compared to CSV86-MEC1 (Fig. 4). This can be attributed to the enhanced transport of Carbaryl in CSV86-MEC2, facilitated by the McbT transporter. *P. bharatica* CSV86^T^ (negative control) failed to utilize and grow on Carbaryl (tested till 48 h) as it lacks CH and 1NH (Table 3; Fig. 4). *Pseudomonas* sp. C5pp displayed faster growth (μ = 0.27 h^−1^) on Carbaryl than engineered strains of CSV86^T^. The total biomass obtained for strain C5pp on Carbaryl (0.1% [wt/vol]) was comparable to CSV86-MEC1 and CSV86-MEC2 (1.6–1.7 g wet weight·L^−1^). The lower specific growth rate of CSV86-MEC1 or CSV86-MEC2 could probably be due to the absence of global adaptive responses, such as oxidative stress response, general stress response, changes in membrane-fatty acid composition, and overall energy regulation (55) required for the efficient metabolism of Carbaryl. The strain C5pp isolated from soil with the ability to degrade Carbaryl would have gone through evolution and adaptation steps/process to optimize the specific growth rate (7, 41). CSV86-MEC2 exhibited good growth (OD_540_ = 0.37 at 12 h) on 1-naphthol (0.01% [wt/vol]) in the presence of yeast extract (0.025% [wt/vol]) with characteristic olive-green color. Whereas, wild-type CSV86^T^ (negative control) failed to grow on 1-naphthol even in the presence of yeast extract, as it lacks 1NH.

**Fig 5:**
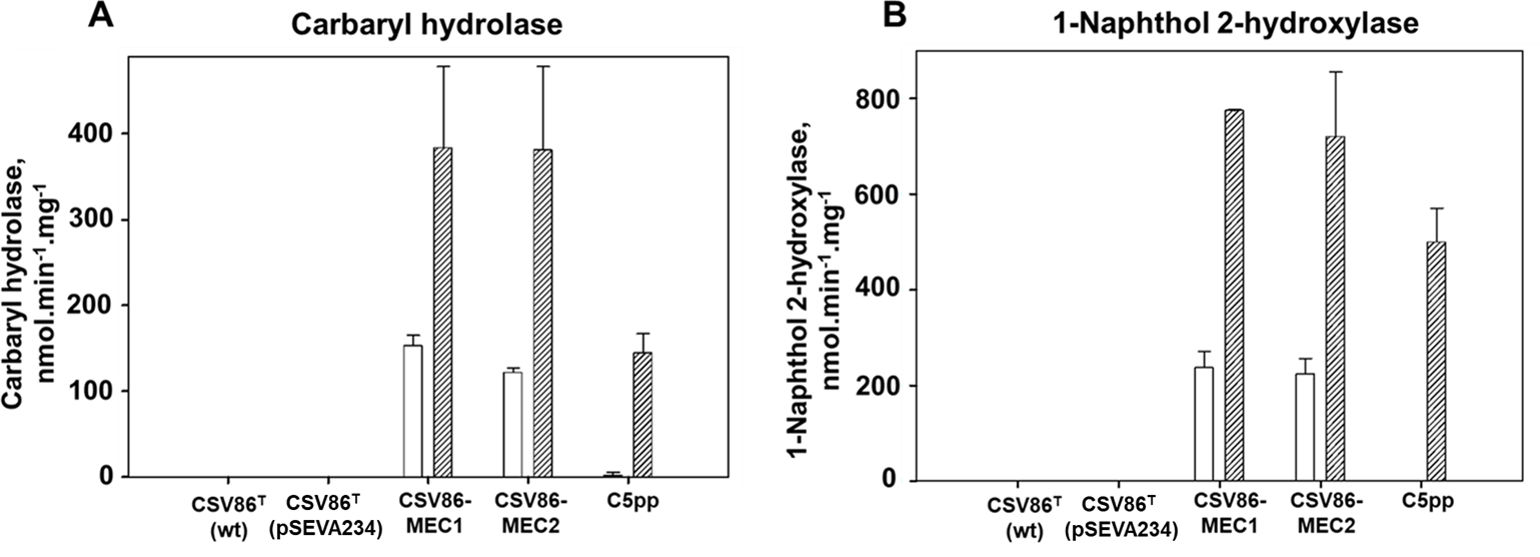
Specific activity of (**A**) Carbaryl hydrolase and (**B**) 1-naphthol 2-hydroxylase in CSV86-MEC1, CSV86-MEC2, and strain C5pp grown on minimal salt medium supplemented with glucose (open bar) or Carbaryl (dashed bar). The mean values of at least three independent experiments performed in triplicates with standard deviation are depicted.

The activities of CH and 1NH as well as C23DO (encoded by *nah*H gene of the *sal* operon from strain CSV86^T^; transcribed by P*sal* promoter) were monitored from Carbaryl- and glucose-grown cells of CSV86-MEC1 and CSV86-MEC2. These analyses were conducted to assess the induction of degradative pathway enzymes by Carbaryl and its metabolism through the salicylate-catechol route. Naphthalene-grown strain CSV86^T^ or it carrying pSEVA234 (parent vector) showed negligible CH and 1NH activity (3–4 nmol min^−1^ mg^−1^; Fig. 5). Whereas, CH (2.5 nmol·min^−1^·mg^−1^) and 1NH (not detectable) activity in glucose-grown strain C5pp was negligible and displayed induction in Carbaryl-grown cells (CH: 145 ± 22 nmol·min^−1^·mg^−1^; 1NH: 500 ± 71 nmol·min^−1^·mg^−1^; Fig. 5). Carbaryl-grown CSV86-MEC1 and CSV86-MEC2 cells showed ~2.5- to 3-fold higher activity of CH (384 ± 94 nmol·min^−1^·mg^−1^ for CSV86-MEC1; 381 ± 97 nmol·min^−1^·mg^−1^ for CSV86-MEC2) and ~3.2-fold higher activity of 1NH (775 ± 3 nmol·min^−1^·mg^−1^ for CSV86-MEC1; 720 ± 135 nmol·min^−1^·mg^−1^ for CSV86-MEC2) as compared to glucose-grown cells (CH: 154 ± 11 nmol·min^−1^·mg^−1^ for CSV86-MEC1 and 122 ± 5 nmol·min^−1^·mg^−1^ for CSV86-MEC2; 1NH: 238 ± 34 nmol·min^−1^·mg^−1^ for CSV86-MEC1 and 224 ± 32 nmol·min^−1^·mg^−1^ for CSV86-MEC2; Fig. 5). Thus, the upregulation/induction of CH and 1NH (expressed under P*nah*) confirmed the metabolism of Carbaryl to 1,2-dihydroxynaphthalene via 1-naphthol, which is subsequently metabolized to salicylate. The P*nah* promoter is reported to be activated by salicylate upon binding to the NahR transcriptional activator (38).

The activity of C23DO (a part of the CSV86^T^ genome-encoded naphthalene lower pathway) was observed to be ~1,100 nmol·min^−1^·mg^−1^ in the CFE of CSV86-MEC1 and CSV86-MEC2 cells grown on Carbaryl, which is ~10- to 16-fold higher than the glucose-grown cells (~70–100 nmol·min^−1^·mg^−1^). This activity is comparable to the previously reported C23DO activity of glucose-grown CSV86^T^ (~150 nmol min^−1^ mg^−1^ [30]). C23DO is encoded by the *nah*H gene of the *sal* operon (transcribed through P*sal* promoter), which is activated by salicylate-bound NahR transcriptional activator protein. Thus, induction of C23DO activity in Carbaryl-grown CSV86-MEC2 suggests metabolism via salicylate-catechol route. The strain CSV86^T^ metabolizes catechol either via *ortho*-cleavage (catechol-1,2-dioxygenase [C12DO]; benzoate metabolic pathway) or *meta*-cleavage (C23DO; naphthalene/salicylate metabolic pathway) route. Carbaryl-grown CSV86-MEC2 showed negligible activity of C12DO (~12 nmol min^−1^ mg^−1^, *ortho* ring-cleaving enzyme), suggesting that catechol generated from Carbaryl is metabolized through the *meta* ring-cleavage route. In CSV86^T^, C12DO is encoded by the *cat*A gene, part of *ben* operon, responsible for benzoate degradation and is induced by benzoate (37, 56).

The induction of the naphthalene pathway (*nah* and *sal* operon) by Carbaryl was analyzed by measuring the cell respiration rates (*in vivo*) on various pathway intermediates using whole cells grown on single substrate, i.e., Carbaryl, naphthalene, or glucose as the sole carbon source (Table 4). Carbaryl-grown cells of CSV86-MEC2 showed oxygen consumption (9 nmol·min^−1^·mg^−1^) on Carbaryl which was similar to Carbaryl-grown C5pp (~7 nmol·min^−1^·mg^−1^). Wild-type CSV86^T^ showed negligible respiration on Carbaryl as it lacks CH and 1NH (Table 4). Interestingly, naphthalene-grown wild-type CSV86^T^ cells displayed oxygen uptake on 1-naphthol (~28 nmol·min^−1^·mg^−1^), which could be probably due to naphthalene dioxygenase, reported to have a broad substrate specificity (15, 33, 37). Compared to naphthalene-grown wild-type CSV86^T^ or CSV86-MEC2, the oxygen consumption by Carbaryl-grown CSV86-MEC2 cells on naphthalene or catechol was low (~0.5- to 0.8-fold, Table 4). However, the oxygen uptake rates on salicylate were comparable. The oxygen uptake rates of Carbaryl-grown CSV86-MEC2 on naphthalene, salicylate, and catechol were ~2.5-, 3.9-, and 3.7-fold higher, respectively, than glucose-grown cells of CSV86-MEC2 (Table 4). *Pseudomonas* sp. C5pp was the only strain which exhibited oxygen consumption on gentisate, as it serves as a metabolic intermediate in the Carbaryl degradation pathway of the strain. Furthermore, the strain C5pp exhibits the ability to utilize gentisate as sole carbon source (7). Whereas, strain CSV86^T^ (wild type or engineered) does not grow or show oxygen consumption on gentisate as it lacks gentisate degradation enzymes.

**TABLE 4:** Whole-cell O_2_ uptake by *Pseudomonas* sp. C5pp, *P. bharatica* CSV86^T^, CSV86-MEC1, and CSV86-MEC2 cells on various metabolic intermediates

Organism (grown on^*a*^)	Whole-cell oxygen uptake (nmol O_2_ consumed min^−1^ mg^−1^ of cells) on metabolic intermediates					
	Carbaryl	1-Naphthol	Naphthalene	Salicylate	Catechol	Gentisate
*Pseudomonas* sp. C5pp (Carbaryl)	6.6 ± 0.9	22 ± 6.8	Tr^*b*^	3.2 ± 1.1	Tr^*b*^	2.4 ± 0.7
*P. bharatica* CSV86^T^ (naphthalene)	0.8 ± 0.1	28 ± 3.6	50 ± 7	3.1 ± 0.8	41 ± 3	Tr^*b*^
CSV86-MEC1 (Carbaryl)	7.4 ± 0.4	16 ± 0.6	24 ± 1.5	4.6 ± 0.3	26 ± 2.5	Tr^*b*^
CSV86-MEC2 (Carbary)	9 ± 3	13 ± 4.6	23 ± 2.6	5.8 ± 2.6	27 ± 0.2	Tr^*b*^
CSV86-MEC2 (naphthalene)	9 ± 2.2	27 ± 3.1	42 ± 4	4.5 ± 1.1	33 ± 3	Tr^*b*^
CSV86-MEC2 (glucose)	6.4 ± 3.6	17 ± 0.6	9.2 ± 1	1.5 ± 0.4	7.3 ± 0.3	Tr^*b*^

^*a*^ Cells were grown on minimal salt medium containing respective aromatic compound or glucose as the sole carbon source.

^*b*^ Tr, trace; whole-cell oxygen consumption less than 0.5 nmol min^−1^ mg^−1^ is reported as trace.

Thus, Carbaryl could induce the host naphthalene pathway as the oxygen uptake rates on various metabolic intermediates were increased in Carbaryl-grown cells as compared to glucose-grown cells. Carbaryl-grown CSV86-MEC1 exhibited oxygen uptake rates similar to CSV86-MEC2 (Table 4). Thus, the expression of McbT, in addition to CH and 1NH, did not have an impact on cell respiration of the engineered strains.

The whole-cell biotransformation of Carbaryl by CSV86-MEC2 cells was carried out to identify the metabolic intermediates by thin layer chromatography (TLC). The spent medium analysis at various time points during biotransformation showed spots corresponding to 1-naphthol (*R*_*f*_ = 0.75; reddish brown quench), salicylate (*R*_*f*_ = 0.36; sky blue fluorescence), catechol (*R*_*f*_ = 0.39; deep purple quench), and Carbaryl (*R*_*f*_ = 0.60; dark blue quench) with maximum intensity at 6 h, which decreased majorly by 9 h (Fig. S5). The presence of 1-naphthol, salicylate, and catechol in the spent medium indicated that Carbaryl is metabolized through the salicylate-catechol route in CSV86-MEC2.

These metabolic studies (enzyme activity, whole-cell respiration, and biotransformation) indicate that the engineered CSV86^T^ metabolizes Carbaryl to 1,2-dihydroxynaphthalene via 1-naphthol (by CH and 1NH), which is further converted to catechol via salicylate. Generated catechol is funneled through *meta* ring-cleavage route to the central carbon pathway.

### Carbaryl degradation phenotype is stable in *P. bharatica* CSV86^T^

The Carbaryl degradation phenotype of CSV86-MEC2 (encoded by pMEC2) was found to be stable (96%–100%) in the absence of selection pressure (kanamycin or Carbaryl) for cells grown on lysogeny broth (LB) for 96 h or repeatedly sub-cultured for 15 transfers (equivalent to 75–90 generations). Furthermore, randomly selected colonies showed CH (~250–350 nmol·min^−1^·mg^−1^) and 1NH (~800–1,000 nmol·min^−1^·mg^−1^) activity, corroborating the stability data. Similar results were obtained for pMEC1. The loss of plasmid in the absence of selection pressure poses a major disadvantage for bioremediation and metabolic engineering applications. However, pMEC1 or pMEC2 encoding Carbaryl degradation enzymes was stable in strain CSV86^T^ in the absence of selection pressure, indicating suitability for bioremediation applications.

### Engineered CSV86-MEC2 prefers Carbaryl over glucose

Wild-type strain CSV86^T^ metabolizes aromatic compounds preferentially over glucose and co-metabolizes them with organic acids, a unique property that makes strain extremely desirable for bioremediation (28–30). To assess the preferential utilization phenotype of CSV86-MEC2, growth kinetics and metabolic studies were performed on mixed carbon source, Carbaryl plus glucose or naphthalene plus glucose.

CSV86-MEC2 on naphthalene plus glucose (using naphthalene inoculum) displayed diauxic growth profile with utilization of naphthalene in the first exponential phase (Fig. S6). The observed growth profile and culture properties of CSV86-MEC2 were similar to that of wild-type CSV86^T^ (29), and the presence of the plasmid did not impact the preferential utilization phenotype.

**Fig 6:**
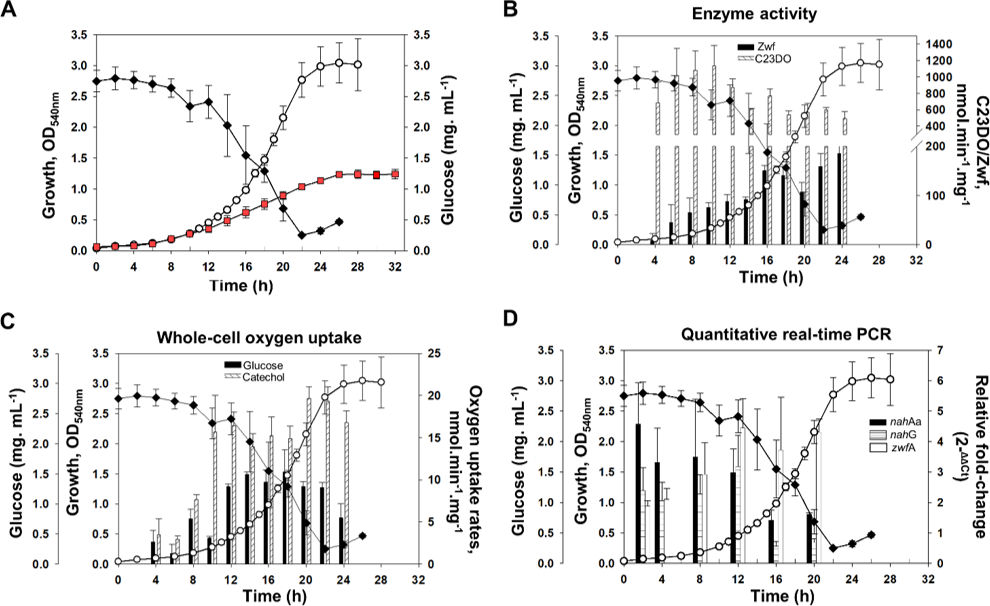
Growth profile and metabolic properties of CSV86-MEC2 on double carbon source: (**A**) Growth profile on Carbaryl (0.1%) (■), or Carbaryl (0.1%) plus glucose (0.25%) (**◯**). The extracellular concentration of glucose from the medium is indicated by ♦. (**B**) Growth-dependent specific activity profile of catechol-2,3-dioxygenase and glucose-6-phosphate dehydrogenase (Zwf) on Carbaryl plus glucose. The C23DO activity is indicated by dashed bars while Zwf by solid bars. (**C**) Growth-dependent whole-cell oxygen uptake profile on Carbaryl plus glucose. The oxygen uptake on catechol at various time points is indicated by dashed bars while on glucose by solid bars. (**D**) Growth-dependent quantitative real-time PCR profile of the genes *nah*Aa, *nah*G, and *zwf*A on Carbaryl plus glucose. The relative fold change (2^−ΔΔCt^) for *nah*Aa is indicated by black solid bars, *nah*G by dashed bars, and *zwf*A by open bars. Values are corrected for *rpo*D as internal reference. The mean value of at least three independent experiments performed in triplicates with standard deviation is depicted.

On Carbaryl plus glucose, CSV86-MEC2 exhibited a growth profile with first lag phase of 6–8 h and second lag phase (small deflection, not so distinct) at 17–18 h (Fig. 6A). The observed growth rates were 0.20 h^−1^ for the first and 0.16 h^−1^ for the second log phase. The extracellular concentration of glucose was steady till 12 h, after which it decreased linearly till 22 h indicating glucose utilization, which coincides with the second log phase. On Carbaryl alone, CSV86-MEC2 showed slower growth with a specific growth rate of 0.11 h^−1^. The presence of glucose during Carbaryl metabolism might aid in overcoming the toxicity of Carbaryl and 1-naphthol generated, resulting in better growth. Glucose might play a role in providing tolerance to oxidative stress during Carbaryl metabolism in CSV86-MEC2. The glucose metabolic enzyme Zwf has been implicated to play a major role in oxidative stress tolerance in *Pseudomonas* spp. by generation of NAD(P)H (57). Glucose has been reported to mitigate the toxicity and enhance degradation of various xenobiotics such as *p-*nitrophenol (58), 4-chlorophenol (59), 2,4-dichlorophenol (60), and 1-naphthol (61) in bacterial isolates.

To assess the carbon utilization hierarchy, the activities of C23DO (indicator of Carbaryl utilization via catechol) and Zwf (indicator of glucose utilization) from the CFE as well as whole-cell oxygen uptake on catechol and glucose were monitored from CSV86-MEC2 growing on Carbaryl plus glucose. The activity of C23DO was found to be maximum at 6–10 h (~1,100 nmol·min^−1^·mg^−1^, in the first log-phase) of diauxic growth profile (Fig. 6B). The activity of Zwf was observed to increase gradually reaching maximum at 16–18 h (~150 nmol·min^−1^·mg^−1^, in the second log phase), which correlates with the linear decrease in the extracellular concentration of glucose (Fig. 6B). During the initial phase of growth, cells exhibited gradual increase in the oxygen consumption on catechol, reaching maximum at 10–12 h (~16 nmol·min^−1^·mg^−1^; Carbaryl utilization phase; Fig. 6C). Whereas, cells displayed maximum respiration on glucose at 16–18 h (~11 nmol·min^−1^·mg^−1^; glucose utilization phase; Fig. 6C). The growth, enzyme activity, and whole-cell oxygen uptake profiles suggest Carbaryl utilization during 4–14 h followed by glucose utilization.

To assess the induction of Carbaryl/glucose metabolic genes in CSV86-MEC2 on Carbaryl plus glucose, qPCR analysis was performed at various time points. The fold-change of genes *nah*Aa (naphthalene dioxygenase reductase subunit; part of *nah* operon; transcribed by P*nah*) and *nah*G (salicylate-1-hydroxylase; part of *sal* operon; transcribed by P*sal*) was measured as indicators of Carbaryl metabolism. While fold-change of *zwf*A (glucose-6-phosphate dehydrogenase) was assessed as indicator of glucose metabolism. The qPCR analysis indicated that the Carbaryl degradation genes were induced maximally (~4- to 5-fold for *nah*Aa and approximately ~2- to 3-fold for *nah*G) at 2–8 h (first log phase), whereas *zwf*A showed maximum induction at 12–20 h (~5-fold increase; second log phase; Fig. 6D) which corroborates with glucose consumption, enzyme activity, and oxygen uptake analyses of the diauxic growth profile.

Carbaryl degradation metabolites from the spent media of CSV86-MEC2 while growing on Carbaryl plus glucose were extracted and analyzed by TLC and high-performance liquid chromatography (HPLC) to assess carbon source utilization. TLC analysis showed metabolite spot corresponding to salicylate (*R*_*f*_ = 0.29; sky blue fluorescence) as early as 4 h (Fig. S7). HPLC analysis showed metabolite peaks corresponding to salicylate and 1-naphthol at 4 h (Fig. S8). The presence of 1-naphthol and salicylate during the early (at 4 h) phase of the growth suggests that the presence of glucose does not suppress Carbaryl utilization in the engineered strain CSV86-MEC2.

On Carbaryl plus succinate, CSV86-MEC2 exhibited a monophasic growth profile, indicating co-metabolism (Fig. S9). Thus, succinate does not repress Carbaryl metabolism in CSV86-MEC2. This is advantageous for bioremediation since in strain C5pp, the utilization of Carbaryl is strongly repressed by the presence of glucose or succinate (62). The observed carbon utilization hierarchy is similar to *P. bharatica* CSV86^T^ (29, 30).

### Conclusion

**Fig 7:**
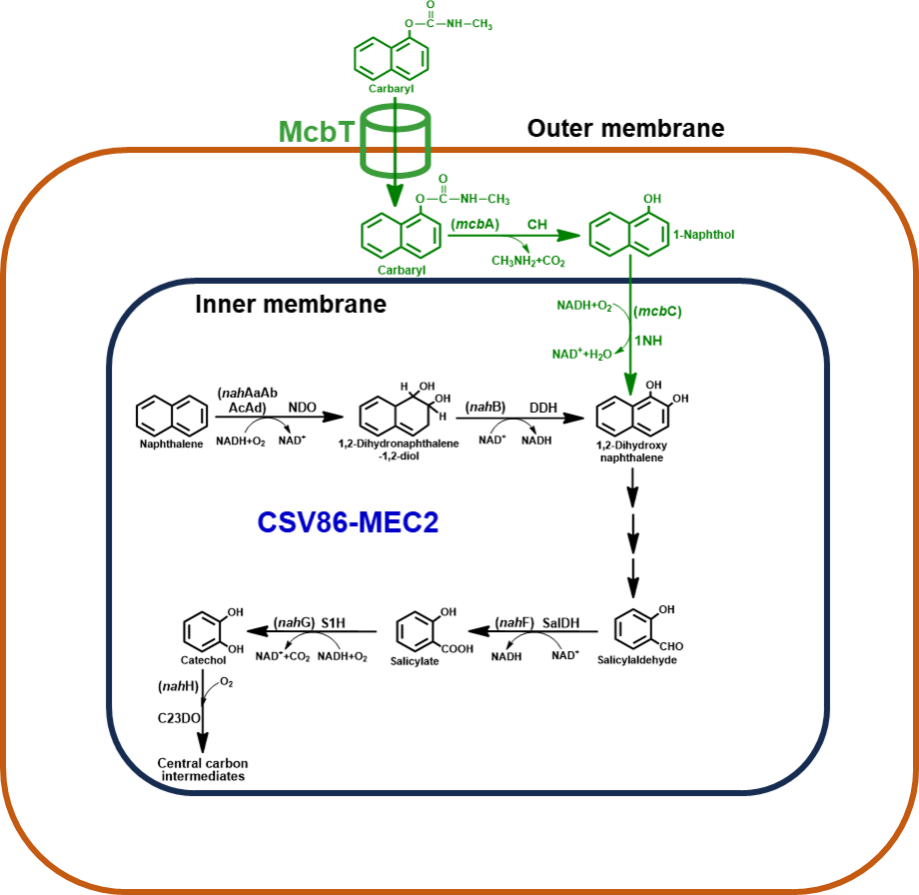
The expression of Carbaryl hydrolase, 1-naphthol 2-hydroxylase, and the putative outer membrane protein McbT (indicated in green) renders CSV86-MEC2 to utilize Carbaryl as the carbon source via salicylate-catechol route.

The co-expression of CH and 1NH resulted in funneling of Carbaryl into the native naphthalene pathway of *P. bharatica* CSV86^T^ (Fig. 7). Furthermore, both enzymes were found to be localized in the periplasm of the engineered strain (pathway compartmentalization), thus preventing the interaction of toxic 1-naphthol with cytoplasmic macromolecules. In addition to CH and 1NH, the expression of McbT (Fig. 7), the putative outer membrane Carbaryl transporter, resulted in increased growth rate of the engineered strain on Carbaryl. However, the growth of strain C5pp was faster as compared to the engineered strain CSV86-MEC2. Over the course of evolution, strain C5pp would have acquired global adaptive responses to the presence of Carbaryl and its metabolites. Adaptive laboratory evolution can further be employed to optimize the growth of the engineered strain CSV86-MEC2 by subculturing on Carbaryl as the sole carbon source. The engineered strain CSV86-MEC2 degraded Carbaryl preferentially over glucose and co-metabolized it with organic acid, offering a major advantage over strain C5pp (which prefers glucose or succinate over Carbaryl) in bioremediation. Overall, metabolic diversity of CSV86^T^ was successfully broadened to degrade naphthalene-based carbamate pesticide Carbaryl, in addition to naphthalene and methylnaphthalenes. Based on these results and stability experiments, we propose that *P. bharatica* CSV86^T^ is an ideal host for the engineering of various aromatic compound metabolic routes for efficient bioremediation. This genetically modified organism (GMO) can be used for the clean-up of Carbaryl- and/or 1-naphthol-contaminated soil or wastewater. However, various ethical/legal issues such as ecological disruption, occurrence of unwanted genetic crossover events/mutations, and horizontal gene transfer of antibiotic selection markers limit the application of GMOs for clean-up of contaminated niches. Therefore, in a controlled environment, a bioreactor-based *ex situ* bioremediation approach that ensures the containment and destruction of the GMO before release of the treated samples into the environment is ideal. Furthermore, immobilized engineered strains can be applied for the bioremediation of contaminated wastewater using constructed wetlands, which can operate in a subsurface flow (vertical flow) or surface flow (horizontal) mode.

## MATERIALS AND METHODS

### Microorganisms, culture conditions, and growth kinetics

*Pseudomonas bharatica* CSV86^T^ (27, 33, 35), *Pseudomonas* sp. C5pp (7), and *Escherichia coli* DH5α (Novagen) were used in the present study (Table 1). Strain CSV86^T^ (wild type or engineered) was grown either on lysogeny broth (63) or 150 mL minimal salt medium (MSM) pH 6.8 (for Carbaryl [7]) or pH 7.5 (for other carbon sources [37]) supplemented with a single-carbon source, such as naphthalene (0.1% [wt/vol]), Carbaryl (0.1% [wt/vol]), 1-naphthol (0.01% [wt/vol]), or glucose (0.25% [wt/vol]), or double-carbon source, such as naphthalene (0.1% [wt/vol]) plus glucose (0.25% [wt/vol]) or Carbaryl (0.1% [wt/vol]) plus glucose (0.25% [wt/vol]) in the absence or presence of kanamycin (40 µg·mL^−1^, for engineered strains) in a baffled Erlenmeyer flask (500 mL) on a rotary shaker (200 rpm) at 30°C. Strain C5pp was grown on 150 mL MSM (pH 6.8) supplemented with Carbaryl (0.1% wt/vol) as a carbon source in a baffled Erlenmeyer flask (500 mL) on a rotary shaker (200 rpm) at 30°C. MSM pH 6.8 was used for growth of various strains on Carbaryl to prevent the autohydrolysis of Carbaryl to 1-naphthol under alkaline conditions. The composition of MSM (pH 7.5) per liter was K_2_HPO_4_ (8 g); KH_2_PO_4_ (1 g); NH_4_Cl (1 g); MgSO_4_·7H_2_O (100 mg); MnSO_4_·H_2_O (1 mg); CuSO_4_·5H_2_O (1 mg); FeSO_4_·7H_2_O (5 mg); H_3_BO_3_ (1 mg); CaCl_2_·2H_2_O (1 mg); NaMoO_4_ (1 mg) (37). The composition of MSM (pH 6.8) per liter was K_2_HPO_4_ (6 g); KH_2_PO_4_ (4.1 g); NH_4_Cl (1 g); MgSO_4_·7H_2_O (100 mg); MnSO_4_·H_2_O (1 mg); CuSO_4_·5H_2_O (1 mg); FeSO_4_·7H_2_O (5 mg); H_3_BO_3_ (1 mg); CaCl_2_·2H_2_O (1 mg); NaMoO_4_ (1 mg) (7). *E. coli* DH5α was used for cloning and plasmid propagation and was grown on LB on a rotary shaker (200 rpm) at 37°C. The growth kinetics of strains were determined by measuring the optical density of the culture at every 1 or 2 h intervals at 540 nm using a spectrophotometer (Lambda-35, PerkinElmer). The specific growth rates (µ) have been calculated using the formula µ = [ln (OD_2_/OD_1_)]/(*t*_2_ − *t*_1_), where OD_2_ denotes the OD_540_ at time point *t*_2_ and OD_1_ denotes the OD_540_ at time point *t*_1_ (64).

### Bioinformatic analyses

#### Sequence analysis

The draft genome sequences of *Pseudomonas* sp. C5pp (GenBank accession number JWLN00000000.1 [65]) and Supercontig-A (GenBank accession number KU522233 [41]) are available at NCBI (https://www.ncbi.nlm.nih.gov/). The Carbaryl upper pathway operon in Supercontig-A of strain C5pp was re-annotated using RAST server (66; https://rast.nmpdr.org/) to reassign the open reading frame *mcb*T located in between *mcb*A and *mcb*B. Promoter prediction was performed using BPROM, and a linear discriminant function score was assigned to each promoter (67; http://www.softberry.com/all.html). An LDF score is an indicator of the promoter strength (a predicted promoter has a minimum LDF score of 0.2, indicating the presence of a σ^70^ promoter with 80% accuracy/specificity) and is assigned by the BPROM server (online promoter prediction tool). The amino acid sequence of McbT was used for signal peptide prediction using SignalP 6.0 (68; https://services.healthtech.dtu.dk/service.php?SignalP-6.0) and secondary structure prediction using PSI-PRED 4.0 (69; http://bioinf.cs.ucl.ac.uk/psipred/).

#### Molecular modeling

The three-dimensional structure of McbT was predicted using ColabFold (https://colab.research.google.com/github/sokrypton/ColabFold/blob/main/AlphaFold2.ipynb; 70) based on AlphaFold 2.0 (71) and MMseqs2 (72). Model with best predicted local distance difference test (pLDDT) score (>90) and least outliers in the Ramchandran plot was selected for further study. PyMOL 2.4.0 (The Molecular Graphics System, Schrödinger, LLC.) was used to visualize and draw protein structure models.

### Molecular biology techniques

#### Cloning of *mcbA* and *mcbT* into pSEVAP*nah*-1NH

The plasmid pSEVAP*nah*-1NH (derived from pSEVA234) expressing 1-naphthol 2-hydroxylase under the P*nah* promoter (which drives the transcription of the naphthalene degradation upper operon; *nah* operon) of CSV86^T^ (38) was used in the current study. The gene *mcb*A (2.3 kb) encoding Carbaryl hydrolase (CH carrying Tmd + Sp at the N-terminus [42] and 6× His tag at C-terminus added using reverse primer CH-RP) was PCR amplified from the genomic DNA of *Pseudomonas* sp. C5pp using primers CH-FP and CH-RP (Table 2). The amplicon was gel eluted, double digested with *Kpn*I/*Bam*HI, and ligated at *Kpn*I/*Bam*HI sites of pSEVAP*nah*-1NH to generate construct pSEVAP*nah*-CH-1NH, referred to as pMEC1 (7.3 kb; Fig. S2A). Similarly, the gene *mcb*T (0.897 kb; encoding full-length McbT, including signal peptide) was amplified using primers, *mcb*T-FP and *mcb*T-RP (Table 2), and cloned at *Bam*HI/*Xba*I sites of pMEC1 to generate construct pSEVAP*nah*-CH-McbT-1NH, referred to as pMEC2 (8.2 kb; Fig. S2B). Constructs were confirmed by gene-specific PCR and Sanger sequencing method (1st BASE, Malaysia). Strain CSV86^T^ was transformed with the construct pMEC1 or pMEC2 by electroporation in the presence of sucrose (300 mM) and glycerol (10%) as described (73). The transformants were selected on LB-agar supplemented with kanamycin (40 µg·mL^−1^). *P. bharatica* CSV86^T^ transformed with pMEC1 is referred as CSV86-MEC1, while CSV86^T^ transformed with pMEC2 is referred as CSV86-MEC2 throughout the study. The P*nah* promoter drives the transcription of CH and 1NH (pMEC1) or CH, McbT, and 1NH (pMEC2) as a polycistronic mRNA.

#### Co-transcription of Carbaryl upper pathway operon

Total RNA was isolated from *Pseudomonas* sp. C5pp cells grown till mid-log phase (OD_540_ = 0.6) on MSM supplemented with Carbaryl using protocol provided with RNeasy Mini Kit (Qiagen, Germany). RNA was made DNA free by treating it with Ambion Turbo RNase-free DNase (Thermo, USA) at 37°C for 60 min. cDNA was synthesized using DNA-free total RNA as template and random hexamers using SuperScript III first-strand synthesis system as per manufacturer’s instructions (Invitrogen, USA). The cDNA was used as template to perform co-transcription analysis of “Carbaryl upper pathway operon” genes using primers listed in Table 2. RNA preparation treated with RNase-H was used as template for PCR used in co-transcription analysis as negative control to assess genomic DNA contamination.

#### Quantitative PCR

The quantification of transcripts of *nah*Aa (naphthalene dioxygenase reductase subunit; part of *nah* operon), *nah*G (salicylate-1-hydroxylase; part of *sal* operon), and *zwf*A (glucose-6-phosphate dehydrogenase) was performed by qPCR for CSV86-MEC2 cells grown on Carbaryl plus glucose at various time points. DNA-free RNA and cDNA were prepared as described above. qPCR was performed using cDNA as a template, gene-specific primers (Table 2), and Platinum SYBR green qPCR SuperMix-UDG with ROX (Invitrogen, Thermo, USA) using StepOnePlus Real-Time PCR system (Applied Biosystems, USA). Primer efficiency was found to be 90%–110% for all genes. Melt curve analysis showed a single peak, while a single band of specific amplicon was detected on agarose gel electrophoresis, indicating amplification was gene specific. RNA preparation treated with RNase-H was used as template for PCR as negative control to assess genomic DNA contamination. The ΔCt value was calculated using *rpo*D (housekeeping gene encoding σ^70^ transcription factor) as an internal reference. The ΔΔCt values were calculated with respect to the basal expression of these genes at 0 h (time of inoculation). Fold change in the gene expression was determined by the mathematical equation 2^−ΔΔCt^ (74).

### Metabolic studies

#### Protein expression, cell-free extract preparation, and Western blot analysis

A single colony of transformants (CSV86-MEC1 or CSV86-MEC2) was inoculated into LB (5 mL) supplemented with kanamycin (40 µg·mL^−1^) and incubated at 30°C on a rotary shaker (200 rpm) for 16 h. Cultures were grown till mid-log phase (OD_540_ = 0.8–1) on MSM (150 mL, pH 7.5) supplemented with naphthalene (12 h) or glucose (16 h) as the sole carbon source in the presence of kanamycin (40 µg·mL^−1^) using LB-grown culture (1 mL) as inoculum. Alternatively, cultures were grown on Carbaryl (150 mL, pH 6.8) using naphthalene-grown culture (2 mL) as inoculum. Strain CSV86^T^ or C5pp was grown on MSM supplemented with naphthalene (8 h) or Carbaryl (12 h), respectively. The cells were harvested by centrifugation at 7,000 × *g* for 10 min (4°C), washed twice, and resuspended (1 g cells in 5 mL) in buffer A (potassium phosphate buffer, 50 mM, pH 7.5). Cells were lysed on ice by sonication (14 W/15 pulses/1 s on, 1 s off/4 cycles) and centrifuged at 30,000 × *g* for 30 min (4°C) to obtain a clear supernatant referred to as cell-free extract, which was used for monitoring various enzyme activities.

Protein concentration in various fractions was determined by the method of Bradford (75) using BSA as the standard. Proteins present in various fractions were analyzed by SDS-PAGE 12%) (76).

To confirm CH expression in various fractions, SDS-PAGE followed by Western blot (77) was developed using anti-histidine primary rabbit antibody (Merck, Germany) and anti-rabbit secondary antibody conjugated with peroxidase (Merck, Germany), and visualized using substrate 3,3′,5,5′-tetramethylbenzidene (Sigma Aldrich, USA).

#### Periplasmic and cytoplasmic fraction preparation

The mid-log phase (OD_540_ = 0.8–1) naphthalene-grown cultures of CSV86-MEC1, CSV86^T^ and Carbaryl-grown culture of strain C5pp were used for the preparation of periplasmic and cytoplasmic fraction using the “cold osmotic shock” method (78). Briefly, cells were harvested by centrifugation at 7,000 × *g* for 10 min (4°C) and washed twice with buffer B (potassium phosphate, 20 mM, pH 7.5). The cells were re-suspended in buffer B (1 g cells in 5 mL) containing MgCl_2_ (0.2 M) and incubated at 35°C for 10 min on shaking water bath followed by incubation on ice for 10 min. Cycle was repeated twice (total three cycles). The periplasmic fraction (supernatant) was obtained by centrifuging the cell suspension at 20,000 × *g* for 20 min at 4°C. The obtained cell pellet was washed twice with ice-cold buffer A, resuspended, and lysed by sonication (as described above). The cell homogenate was centrifuged (30,000 × *g*, 30 min, 4°C), and the obtained supernatant was referred to as “cytoplasmic fraction.” The activities of various enzymes were monitored from these fractions.

#### Enzyme assays

Enzyme activities were monitored spectrophotometrically (Lambda-35, PerkinElmer). The activity of Carbaryl hydrolase was monitored by measuring the rate of appearance of 1-naphthol at 322 nm in buffer A as described previously (7). The activity of 1-naphthol 2-hydroxylase was monitored by measuring the rate of oxidation of NADH at 340 nm in buffer A as described previously (79). The activity of catechol-2,3-dioxygenase was monitored by measuring the rate of appearance of 2-hydroxymuconic semialdehyde at 375 nm in buffer A as described previously (29). The activity of C12DO was monitored by measuring the rate of appearance of *cis,cis*-muconic acid at 260 nm in buffer A as described previously (29). The activity of gentisate-1,2-dioxygenase was monitored by measuring the rate of appearance of maleylpyruvate at 330 nm in buffer A as described previously (7). The activity of glucose-6-phosphate dehydrogenase (Zwf) was monitored by measuring the rate of reduction of NADP^+^ at 340 nm in Tris-Cl buffer (50 mM, pH 7.5) as described previously (80). The total amount of protein used per assay reaction mixture was 25–40 µg. All enzyme activities are expressed as the specific activity in nanomoles of substrate consumed/product formed per minute per milligram of total protein present in the respective fraction (nmol·min^−1^·mg^−1^).

The time-dependent spectral scan for CH reaction to monitor the conversion of Carbaryl to 1-naphthol was recorded spectrophotometrically from 240 to 340 nm at every 1 min interval for 10 cycles. One milliliter reaction mixture contained enzyme (25 µg), Carbaryl (200 µM), and buffer A.

#### Outer membrane protein fractionation

Carbon source-dependent expression of McbT from strain C5pp cells was analyzed by preparing the outer membrane fraction as described by Nakajima et al. (81) with minor modifications. Briefly, cells grown on MSM supplemented with Carbaryl, benzoate, or glucose till late log phase were harvested, washed twice, resuspended (1 g cells in 5 mL) in Tris-Cl buffer (10 mM, pH 8.0, containing 10 mM EDTA), and lysed by sonication. The cell homogenate was centrifuged at 10,000 × *g* for 10 min (4°C) to remove cell debris, followed by ultracentrifugation of supernatant at 50,000 × *g* for 60 min (4°C). The pellet was suspended in *N*-laurylsarcosine (1% in Tris-Cl buffer) and vortexed for 30 min (to dissolve the inner membrane) followed by centrifugation at 50, 000 × *g* for 60 min (4°C). The resulting membrane pellet which consists of outer membrane was suspended in minimum amount of Tris-Cl buffer and analyzed by SDS-PAGE.

#### Whole-cell biotransformation

CSV86-MEC2 was grown till late log phase (OD_540_ = 1.5–1.6) on MSM (150 mL, pH 7.5) supplemented with naphthalene (0.1%; using LB-grown inoculum), washed twice with ice-cold MSM (pH 6.8), resuspended in MSM (50 mL, pH 6.8) supplemented with Carbaryl (0.2% [wt/vol]), and incubated at 30℃ on a rotary shaker at 200 rpm for 0, 3, 6, or 9 h. The spent medium was collected by centrifugation (7,000 × *g*, 10 min, 4°C), acidified to pH 2 with 2 N HCl, and extracted in equal volume of ethyl acetate. The organic phase was dried over sodium sulphate (anhydrous), concentrated, and analyzed by TLC on a silica gel 60 F_254_ (Merck, Germany) using solvent system benzene:acetone:acetic acid (9:1:0.125, vol/vol/vol). Metabolites were identified by comparing the *R*_*f*_ values and UV-fluorescence properties with authentic pure compounds.

#### Spent media analysis

CSV86-MEC2 culture (growing on Carbaryl plus glucose) was centrifuged (7,000 × *g*, 10 min, 4°C) to remove cells, and the supernatant collected was referred to as the spent media. The glucose concentration from the spent medium was estimated by the method of Miller (82) using 3,5-dinitrosalicylic acid reagent. The Carbaryl degradation metabolites were identified after extraction from spent medium in ethyl acetate and resolved by TLC as described above. The metabolites were also resolved and identified by high-performance liquid chromatography with photodiode array detector (HPLC; Jasco, LC-4000) using octadecylsilane silica (C18) reverse-phase column (dimensions: 4.6 × 150 mm). The metabolites were detected at 280 nm (λ_max_ for Carbaryl), 302 nm (λ_max_ for salicylate), and 322 nm (λ_max_ for 1-naphthol). The solvent system used was acetonitrile:water:*ortho-*phosphoric acid (88%) (400:600:2, vol/vol/vol) (83) at a flow rate of 1 mL·min^−1^.

#### Whole-cell oxygen uptake

The whole-cell oxygen uptake was monitored by measuring the rate of oxygen consumption in the presence of various pathway intermediates using Oxygraph (Hansatech, UK) fitted with Clark’s oxygen electrode (29). The late log phase cultures of wild-type or engineered strains grown on various carbon sources were harvested by centrifugation (7,000 × *g*, 10 min, 4°C), washed twice, and resuspended in buffer A (200 mg cells in 1 mL). The reaction mixture (2 mL) contained cells (4 mg), substrate (50 µM), and buffer A. The respiration rates were measured at 30°C and expressed as nanomoles of O_2_ consumed per minute per milligram of cells (nmol·min^−1^·mg^−1^).

#### Plasmid stability

The stability of pMEC2 construct in strain CSV86^T^ (CSV86-MEC2) was assessed. CSV86-MEC2 was grown onto LB (5 mL) in the presence of selection pressure (kanamycin; 40 µg·mL^−1^) for 16 h at 30°C referred to as the zeroth transfer. This was transferred (100 µL) onto fresh LB (5 mL) in the absence of selection pressure (Carbaryl and kanamycin) for 12 h at 30°C and subsequently sub-cultured till 15 transfers (~5–6 generations per transfer) on LB with no selection pressure. In another set of experiment, CSV86-MEC2 was grown on LB in the absence of any selection pressure for 96 h at 30°C. In both the experiments, the retention of kanamycin resistance and Carbaryl degradation phenotype was assessed by replica plate method as well as by measuring CH and 1NH activities from three randomly selected kanamycin-resistant colonies from 0th, 5th, 10th, and 15th transfers or 96 h culture.

## SUPPLEMENTAL MATERIAL

Supplemental figures spectrum.00284-24-s0001.docx Fig. S1-S9.

ASM does not own the copyrights to Supplemental Material that may be linked to, or accessed through, an article. The authors have granted ASM a non-exclusive, world-wide license to publish the Supplemental Material files. Please contact the corresponding author directly for reuse.
