# Supplementary material for: Metabolic engineering of Pseudomonas bharatica CSV86T to degrade Carbaryl (1-naphthyl-N-methylcarbamate) via the salicylate-catechol route
Source: Microbiol Spectr. 2024 Jun 13;12(8):e00284-24. doi: 10.1128/spectrum.00284-24 (PMC11302072; doi:10.1128/spectrum.00284-24)
Supplement: Supplemental figures — Fig. S1-S9. [file spectrum.00284-24-s0001.docx]

**Supplementary material**

**(Spectrum00284-24R1)**

**Metabolic engineering of *Pseudomonas bharatica* CSV86^T^ to degrade Carbaryl (1-naphthyl-*N*-methylcarbamate) *via* the salicylate-catechol route**

Harshit Malhotra^1^, Tushar Dhamale^1^, Sukhjeet Kaur^1^, Sravanti T. Kasarlawar^1^ and Prashant S. Phale^1^ *

^1^Department of Biosciences and Bioengineering, Indian Institute of Technology-Bombay, Powai, Mumbai 400 076, India

* Correspondence: [pphale@iitb.ac.in](mailto:pphale@iitb.ac.in)

Running title: Metabolic engineering of *P. bharatica* CSV86^T^

**Figures**


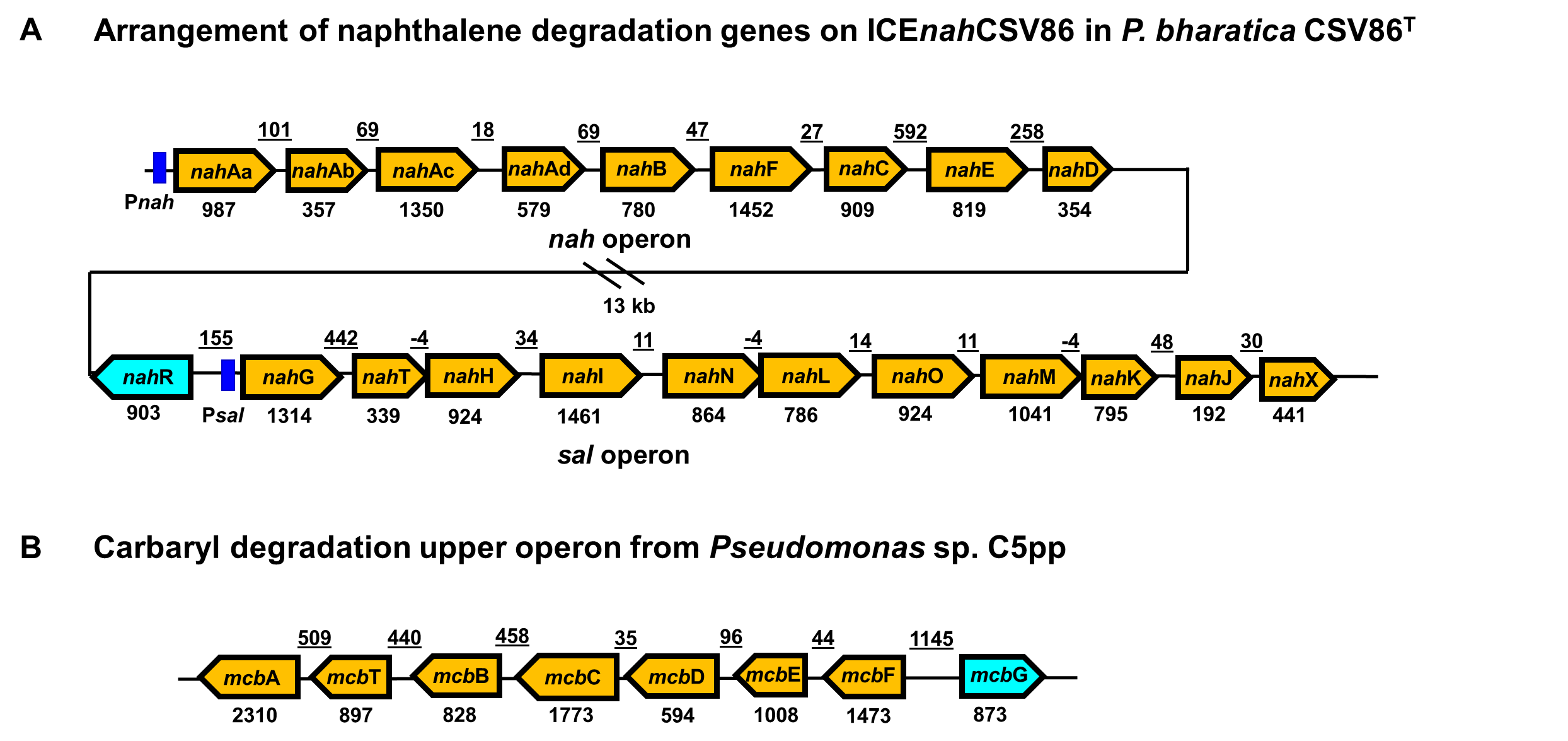


**Figure S1**: Gene arrangement of (A) Naphthalene degradation genes as *nah* and *sal* operons on the integrative and conjugative element ICE*nah*CSV86 in *P. bharatica* CSV86^T^ and (B) Carbaryl degradation upper operon in *Pseudomonas* sp. C5pp. Genes are depicted as orange box arrows with names in italics. The length of the genes and intergenic regions (underlined) are indicated numerically (in bp). Promoters are indicated as dark blue rectangular boxes, while regulatory genes are indicated in cyan box arrows with names in italics.


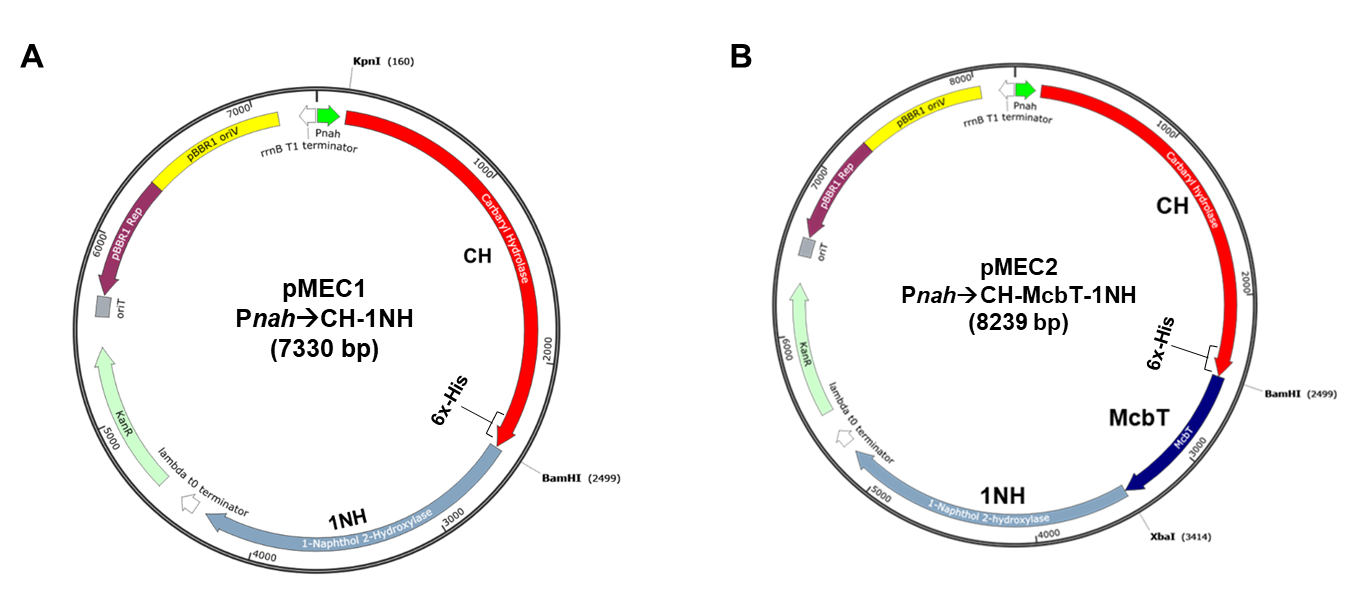


**Figure S2**: Plasmid map of the constructs (A) pMEC1 (P*nah*🡪CH-1NH), encoding Carbaryl hydrolase (CH; harbouring C-terminus 6× His-Tag) and 1-naphthol 2-hydroxylase (1NH) under the P*nah* promoter and (B) pMEC2 (P*nah*🡪CH-McbT-1NH), encoding CH (harbouring C-terminus 6× His-Tag), McbT (putative Carbaryl transporter) and 1NH under the P*nah* promoter. Both vectors have been derived from pSEVA234. The P*nah* promoter has been depicted in green, the gene *mcb*A encoding CH is indicated in red (6×His-Tag at C-terminus), the gene *mcb*C encoding 1NH has been depicted in blue and the gene *mcb*T encoding a putative transporter has been indicated in dark blue. The restriction sites used for cloning have also been indicated.


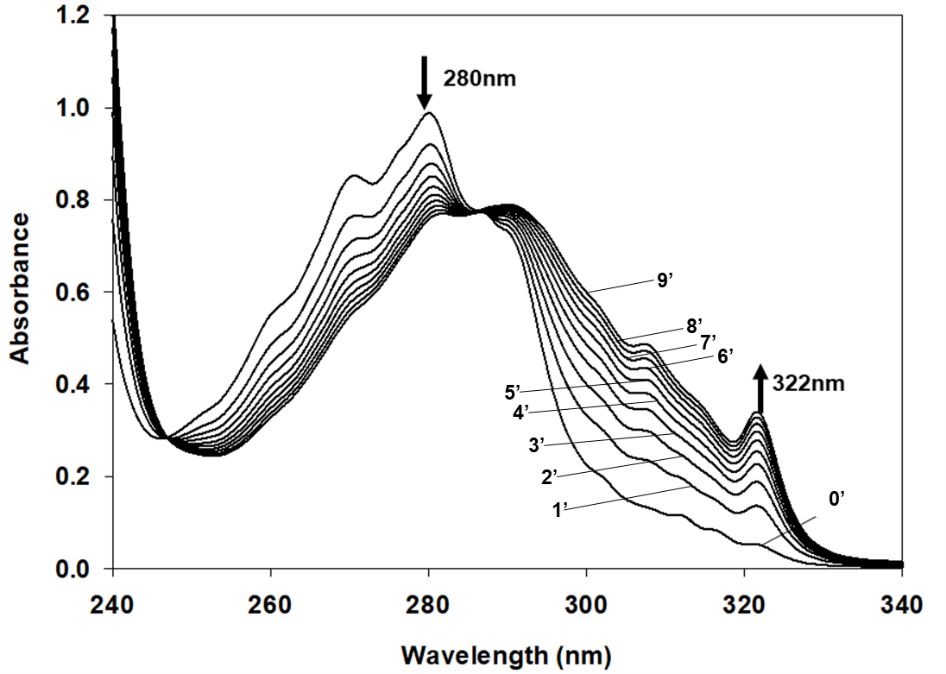


**Figure S3**: Time-dependent spectral changes (10 cycles, 1 min each, 0’-9’) for Carbaryl hydrolase from the periplasmic fraction. Up arrow indicates appearance of 1-naphthol at 322nm, while down arrow indicates the disappearance of Carbaryl at 280 nm.


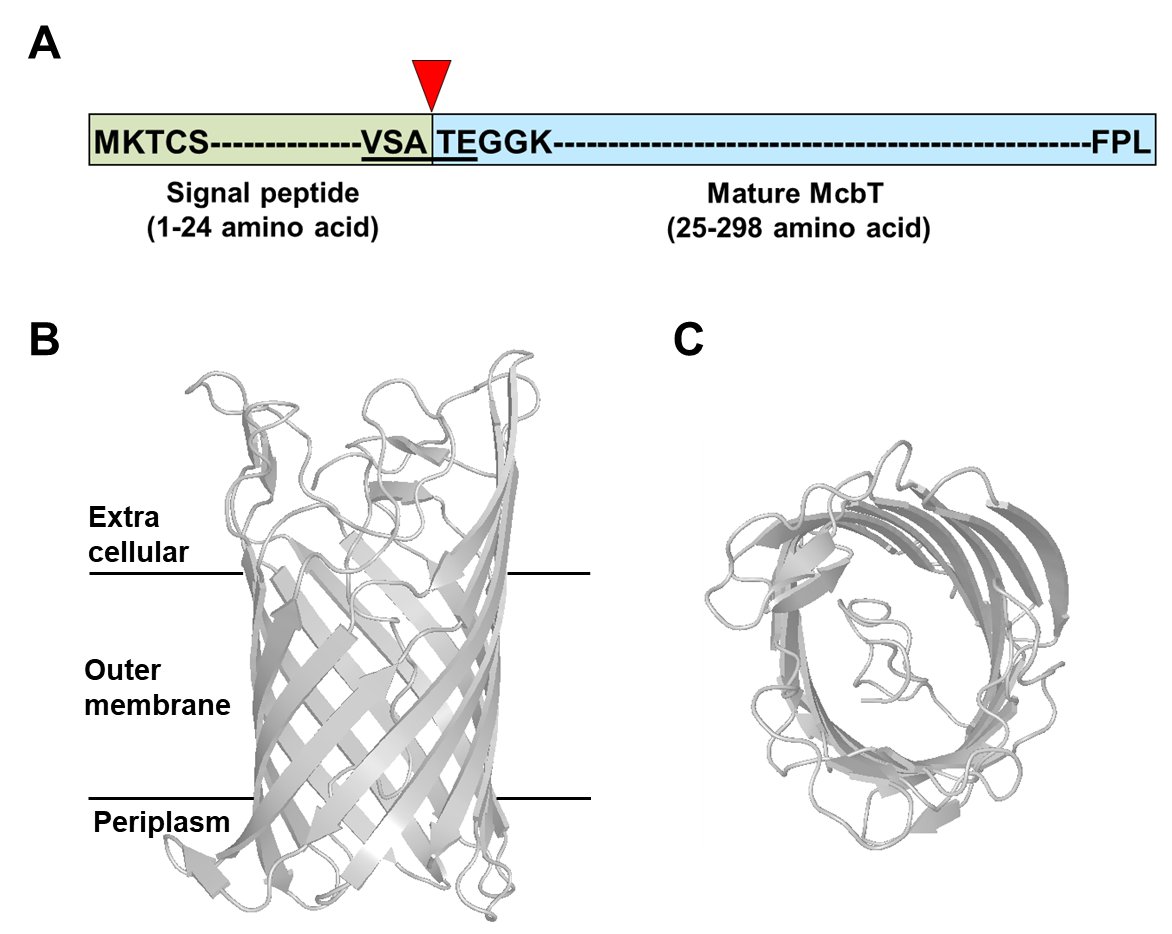


**Figure S4:** Bioinformatic analyses of McbT: (A) Signal peptide and cleavage site prediction of McbT. (B) Three-dimensional structure for McbT using Alphafold2. Cartoon model depicts long loops on the extracellular side and short turns on the periplasmic side. (C) Top view from the extracellular side looking into the barrel lumen. The N-terminus loop occludes the barrel lumen from the periplasmic side.


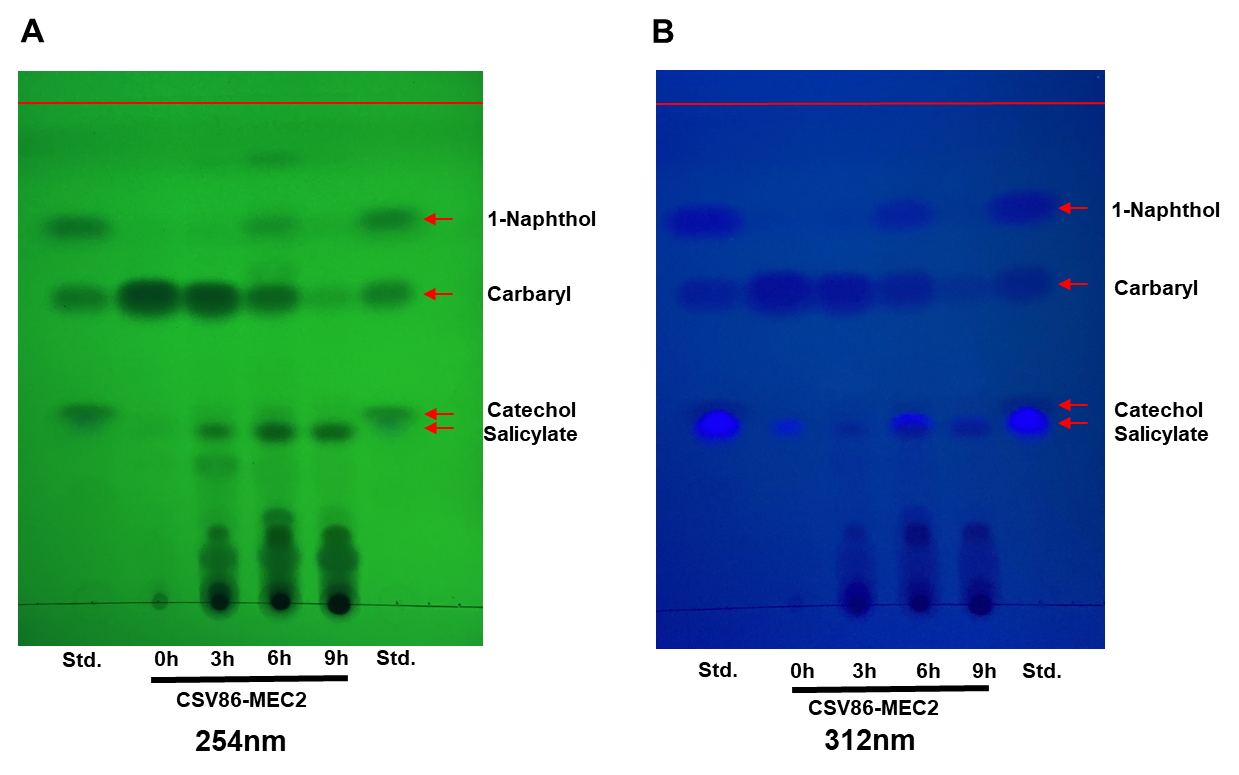


**C**

| **A: Standard** | **R_f_** | **Fluorescence properties at 312 nm** |
| --- | --- | --- |
| Carbaryl | 0.60 | Dark blue quench |
| 1-Naphthol | 0.76 | Reddish brown quench |
| Salicylate | 0.36 | Sky blue fluorescence |
| Catechol | 0.40 | Deep purple quench |
| **B: Metabolites detected from the spent media at 6h of biotransformation** | | |
| Carbaryl | 0.60 | Dark blue quench |
| 1-Naphthol | 0.75 | Reddish brown quench |
| Salicylate | 0.36 | Sky blue fluorescence |
| Catechol | 0.39 | Deep purple quench |

**Figure S5:** Thin layer chromatography (TLC) analysis of whole-cell biotransformation of Carbaryl by CSV86-MEC2. Metabolites were visualized under UV light at (A) 254nm and (B) 312nm. “Std” indicates authentic standards used for analysis, while spots for various metabolites are indicated by red arrows. The solvent front is indicated by a red line (C) R_f_ values and UV fluorescence properties of metabolites generated upon whole-cell biotransformation of Carbaryl by CSV86-MEC2.


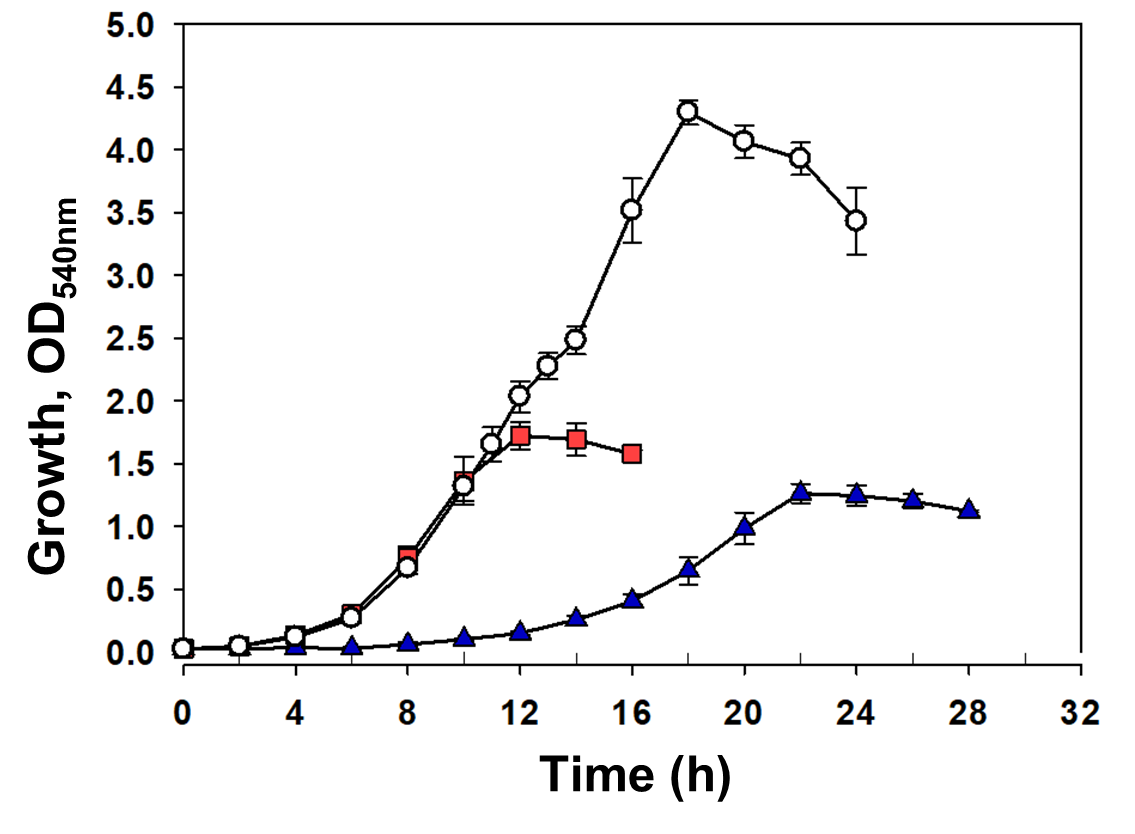


**Figure S6**: Growth profile of CSV86-MEC2 on naphthalene (0.1%) (■), glucose (0.25%) (▲) or naphthalene (0.1%) plus glucose (0.25%) (**◯**).


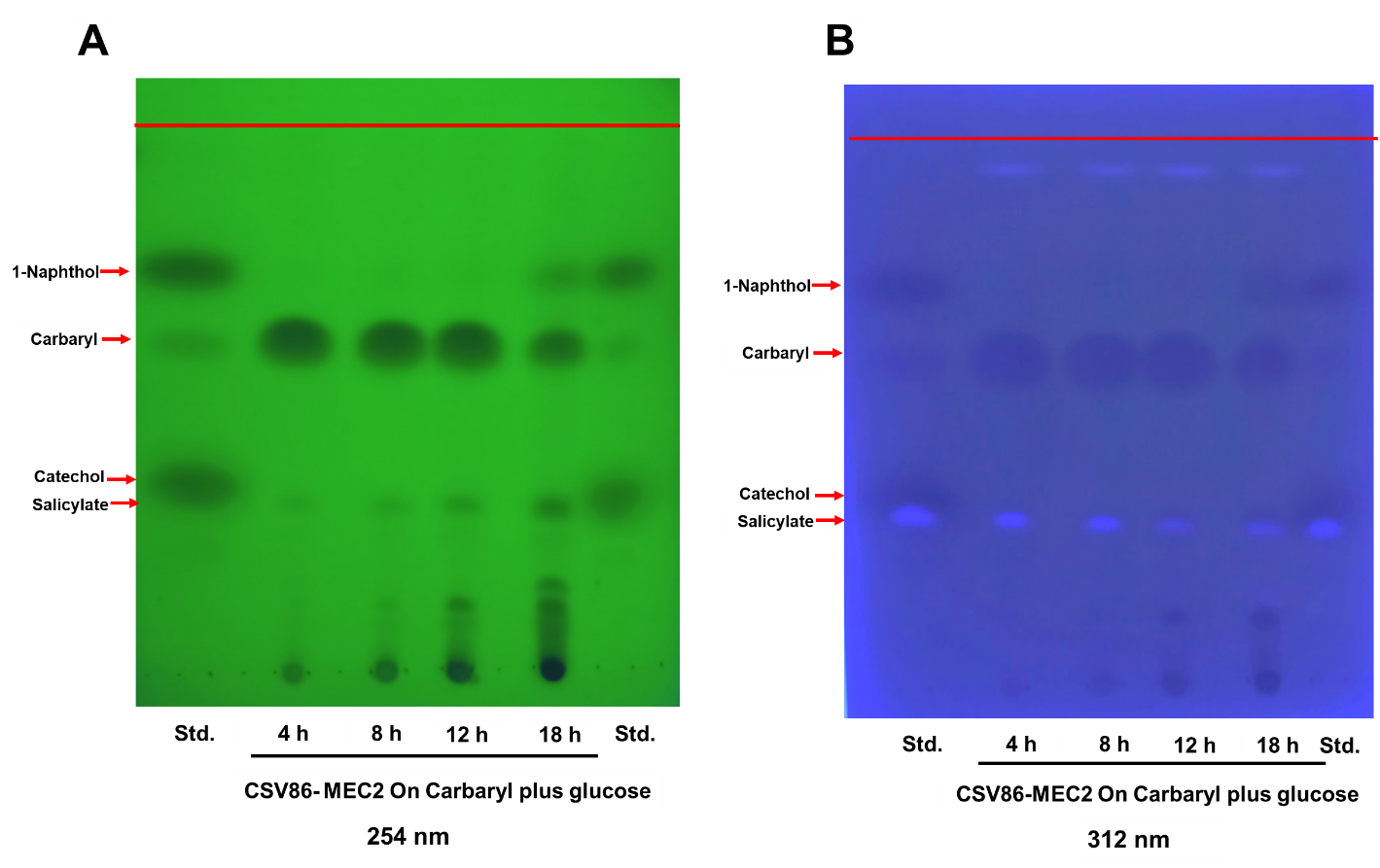


**C**

| **A: Standard** | **R_f_** | **Fluorescence properties at 312 nm** |
| --- | --- | --- |
| Carbaryl | 0.6 | Dark blue quench |
| 1-Naphthol | 0.74 | Reddish brown quench |
| Salicylate | 0.30 | Sky blue fluorescence |
| Catechol | 0.34 | Deep purple quench |
| **B: Metabolites detected in spent media at 18 h of growth on Carbaryl plus glucose** | | |
| Carbaryl | 0.63 | Dark blue quench |
| 1-Naphthol | 0.73 | Reddish brown quench |
| Salicylate | 0.29 | Sky blue fluorescence |
| Catechol | 0.31 | Deep purple quench |

**Figure S7**: Thin layer chromatography (TLC) analysis of spent media of CSV86-MEC2 while growing on Carbaryl plus glucose. Metabolites were visualized under UV light at (A) 254nm and (B) 312nm. “Std.” indicates authentic standards used for analysis, while spots for various standards are indicated by red arrows. The solvent front is indicated by a red line (C) R_f_ values and UV fluorescence properties of metabolites generated by CSV86-MEC2 while growing on Carbaryl plus glucose.

**
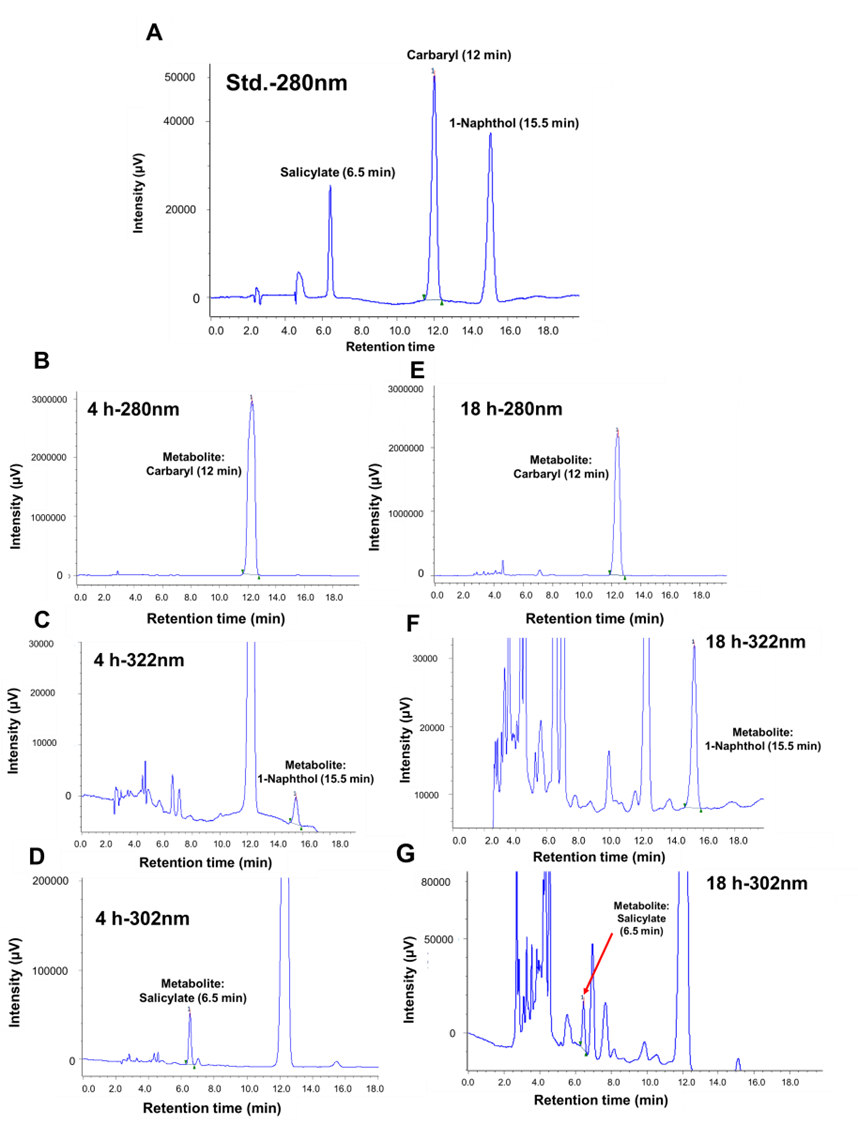
**

**Figure S8**: High-performance liquid chromatography (HPLC) analysis of spent media of CSV86-MEC2 while growing on Carbaryl plus glucose at 4 and 18 hours using acetonitrile:water:orthophosphoric acid (400:600:2; v/v/v) mobile phase. The chromatograms represent (A) mixture of analytical standards of Carbaryl, 1-naphthol and salicylate (500 μM each) at 280 nm (B) 4 h spent media at 280 nm (λ_max_ for Carbaryl) (C) 4 h spent media at 322 nm (λ_max_ for 1-naphthol) (D) 4 h spent media at 302 nm (λ_max_ for salicylate) (E) 18 h spent media at 280 nm (F) 18 h spent media at 322 nm (G) 18 h spent media at 302 nm. The peaks and the retention times have been indicated on the chromatograms.

**
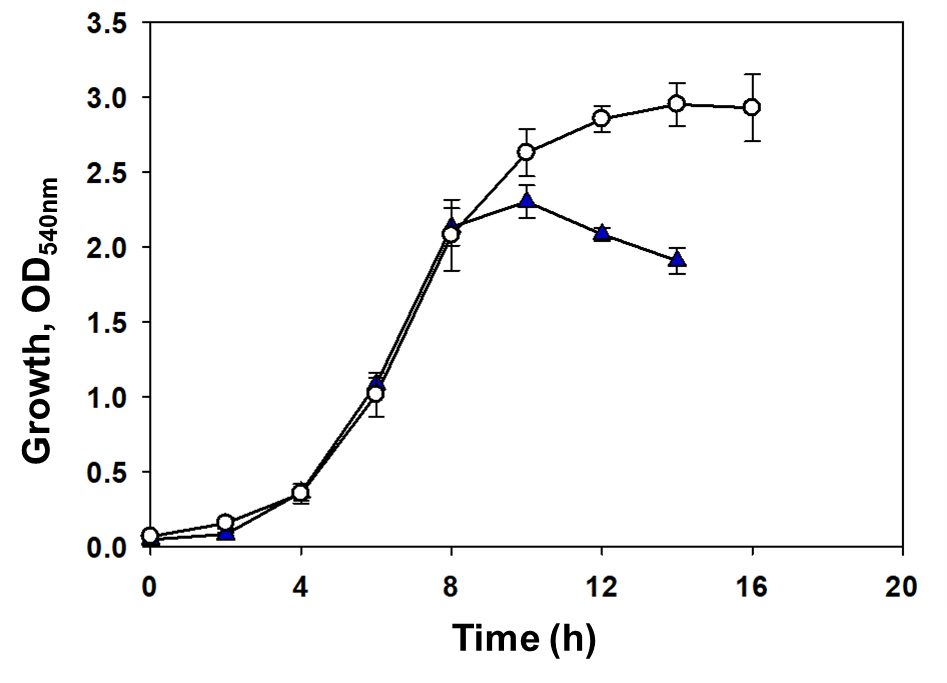
**

**Figure S9**: Growth profiles of CSV86-MEC2 on succinate (0.25%) (▲) or Carbaryl (0.1%) plus succinate (0.25%) (**◯**). The mean value of at least three independent experiments (performed in duplicates) with standard deviation are depicted.
